# Targeting the miR-665-3p-ATG4B-autophagy axis relieves inflammation and apoptosis in intestinal ischemia/reperfusion

**DOI:** 10.1038/s41419-018-0518-9

**Published:** 2018-04-30

**Authors:** Zhenlu Li, Guangzhi Wang, Dongcheng Feng, Guo Zu, Yang Li, Xue Shi, Yan Zhao, Huirong Jing, Shili Ning, Weidong Le, Jihong Yao, Xiaofeng Tian

**Affiliations:** 1 Department of General Surgery, The Second Hospital of Dalian Medical University, 116023 Dalian, China; 2 Department of Pharmacology, Dalian Medical University, 116044 Dalian, China; 3 Clinical Research Center on Neurological Diseases, the First Affiliated Hospital of Dalian Medical University, 116011 Dalian, China

## Abstract

Autophagy is an essential cytoprotective response against pathologic stresses that selectively degrades damaged cellular components. Impaired autophagy contributes to organ injury in multiple diseases, including ischemia/reperfusion (I/R), but the exact mechanism by which impaired autophagy is regulated remains unclear. Several researchers have demonstrated that microRNAs (miRNAs) negatively regulate autophagy by targeting autophagy-related genes (ATGs). Therefore, the effect of ATG-related miRNAs on I/R remains a promising research avenue. In our study, we found that autophagy flux is impaired during intestinal I/R. A miRNA microarray analysis showed that miR-665-3p was highly expressed in the I/R group, which was confirmed by qRT-PCR. Then, we predicted and proved that miR-665-3p negatively regulates ATG4B expression in Caco-2 and IEC-6 cells. In ileum biopsy samples from patients with intestinal infarction, there was an inverse correlation between miR-665-3p and ATG4B expression, which supports the in vitro findings. Moreover, based on miR-665-3p regulation of autophagy in response to hypoxia/reoxygenation in vitro, gain-of-function and loss-of-function approaches were used to investigate the therapeutic potential of miR-665-3p. Additionally, we provide evidence that ATG4B is indispensable for protection upon inhibition of miR-665-3p. Finally, we observed that locked nucleic acid-modified inhibition of miR-665-3p in vivo alleviates I/R-induced systemic inflammation and apoptosis via recovery of autophagic flux. Our study highlights miR-665-3p as a novel small molecule that regulates autophagy by targeting ATG4B, suggesting that miR-665-3p inhibition may be a potential therapeutic approach against inflammation and apoptosis for the clinical treatment of intestinal I/R.

## Introduction

As a severe inflammatory condition, intestinal ischemia-reperfusion (I/R) results from multiple clinical circumstances, such as hemorrhagic shock, sepsis, serious trauma, strangulated intestinal obstruction, and acute mesenteric ischemia, and it leads to an irreversible clinical status with high morbidity and mortality^1–3^. Although intestinal ischemia itself causes irreversible tissue injury, blood restoration is critical for its salvage; however, reperfusion may paradoxically aggravate the tissue damage by inducing apoptotic and inflammatory changes^4,5^. Moreover, inflammatory cytokines in the circulation further induce systemic inflammatory response syndrome (SIRS) and eventually multiple organ dysfunction syndrome (MODS)^6,7^. Therefore, it is of vital importance to elucidate new mechanisms for resisting excessive apoptosis and inflammatory stress to develop innovative therapeutic strategies for treating intestinal I/R.

Autophagy is not only a cellular homeostatic process that is responsible for eliminating damaged protein and organelles but also an adaptive response to provide nutrients and energy upon exposure to various stresses via the lysosomal pathway^8,9^. As a dynamic process, autophagy can be dramatically induced or impaired by multiple stressors. Furthermore, autophagy has a crucial role in cellular and tissue homeostasis through the regulation of inflammation and apoptosis in diverse diseases^10,11^. Reperfusion after cerebral ischemia induces autophagy to alleviate neuronal apoptosis. Interestingly, decreased autophagic flux in hepatic and cardiac I/R injury has been observed, which renders impaired autophagy “inadequate” in preventing cell death^12,13^. Defective autophagy potentiates pro-inflammatory cytokine production and p53 activation^14,15^, while induction of autophagy removes aggregated inflammasome structures and damaged mitochondria^16–18^. Collectively, these studies suggest that autophagy induction may serve as a viable therapeutic strategy. Nevertheless, the specific role of autophagy in intestinal I/R remains undefined.

Autophagy is mediated by complex regulatory networks composed of more than 30 proteins, particularly autophagy-related proteins (ATGs)^19^. Several findings have suggested that ATGs play important roles in maintaining intestinal homeostasis^16,20^. Mutant mice deficient in Atg16L1 with impaired autophagy are highly susceptible to dextran sodium sulfate (DSS)-induced colitis^21^. Reduced ATG5 and ATG16L1 expression after *Escherichia coli* infection leads to an increased inflammatory response^22^. Among the ATGs required for autophagosome formation is ATG4, which is responsible for the activation of pro-ATG8 (mostly the MAP1LC3/LC3 family in yeast) and subsequent conjugation, leading to autophagosome completion^23^. A recent kinetic analysis of all ATG4 orthologs (ATG4A-D) indicated that ATG4B possesses the broadest substrate spectrum and is 1500-fold more catalytically efficient at activating LC3B than the other 3 autophagins^24^. ATG4B is a novel protective protein that plays a crucial role in controlling the inflammatory response during experimental colitis^16^. However, whether ATG4B plays a role in intestinal I/R requires further investigation.

MicroRNAs (miRNAs) are a class of endogenous, non-coding, single-stranded RNA molecules consisting of 22–24 nucleotides, which can be combined with the 3′UTR of target mRNAs to provide post-transcriptional regulation^25^. MiRNAs control various pathophysiological processes, including differentiation, proliferation, and cell death. MiRNAs can act on a variety of ATGs to inhibit their expression, thereby repressing autophagy^26^. Growing evidence has indicated the therapeutic potential of miRNAs in the regulation of ischemic diseases^27,28^. However, it is not yet clear whether miRNAs are involved in regulating autophagy in intestinal I/R injury.

In the present study, we first investigated the potential role of autophagic flux during intestinal I/R. Then, we identified miR-665-3p as a novel autophagy-related miRNA and provided substantial evidence that miR-665-3p regulated the ATG4B mRNA and protein levels in mouse and human samples. Furthermore, miR-665-3p inhibition alleviated the inflammatory response and enterocyte apoptosis during I/R by targeting ATG4B in vivo and in vitro. Taken together, our data describe the role of the miR-665-3p-ATG4B-autophagy pathway in intestinal I/R. Our research implicates miR-665-3p as a potential therapeutic target for ischemic intestinal disease and provides novel insight into miRNA-based therapy.

## Results

### Impaired autophagy during I/R contributes to intestinal injury

**Fig. 1:**
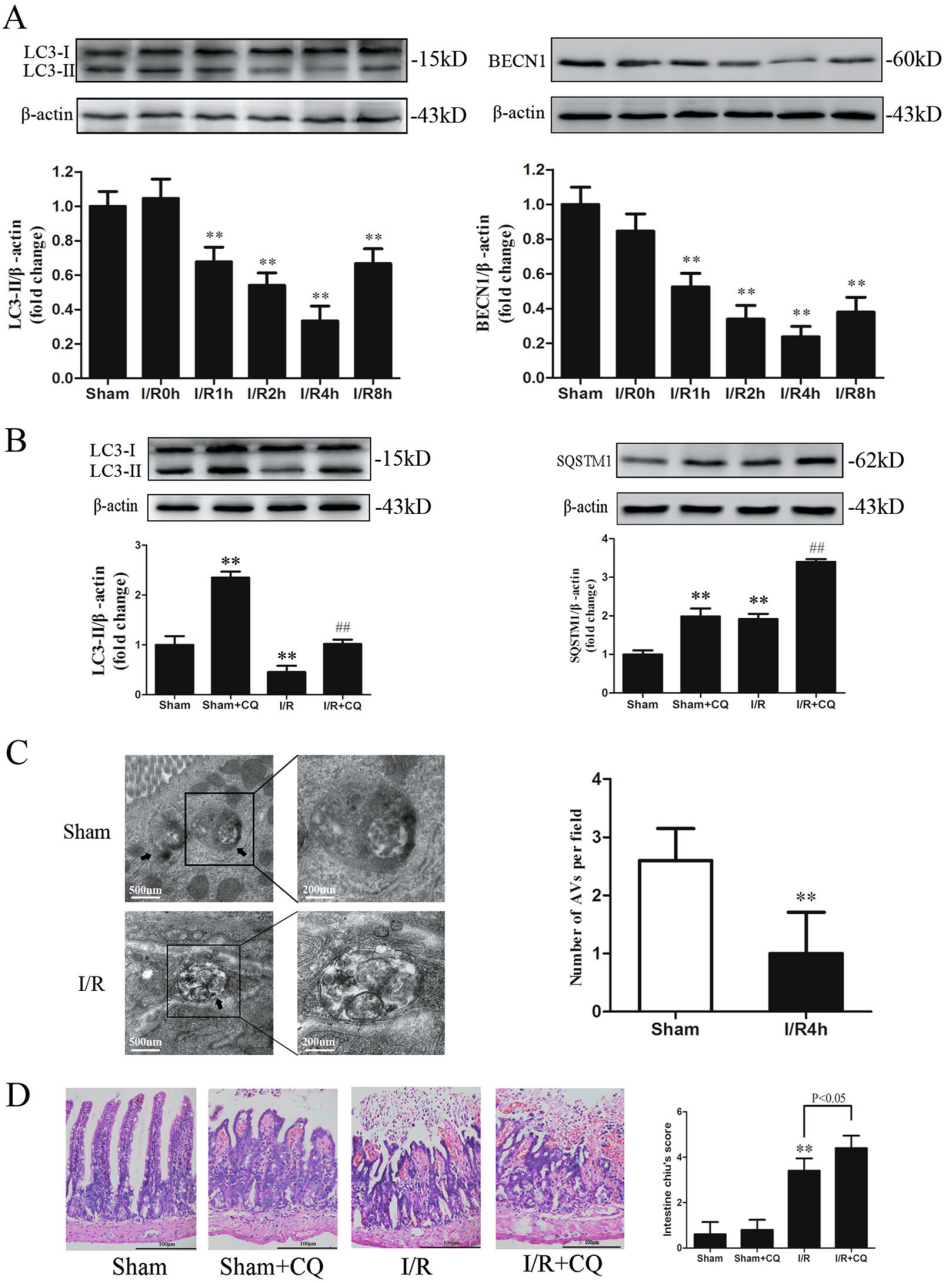
Impaired autophagy during I/R contributes to intestinal injury. **a** Mice were subjected to 45 min of intestinal ischemia, followed by 0–8 h of reperfusion. Representative immunoblots of endogenous LC3 and BECN1 levels in the intestine. **b** LC3 and SQSTM1 levels after 45 min of intestinal ischemia followed by 4 h of reperfusion in the absence and presence of CQ. **c** Representative transmission electron microscopy images and quantitative analysis of AVs in the intestine, *n* = 3. Arrows indicate the AVs. **d** Representative H&E staining and histological injury scores of the gut in the different groups, *n* = 6. ^*^P *<* 0.05, ^**^P *<* 0.01 versus sham. ^##^*P* *<* 0.01 versus sham+CQ

To measure the exact autophagy levels in intestinal I/R, we subjected mice to 45 min of ischemia followed by 0–8 h of reperfusion. The LC3-II and BECN1 levels, which are representative autophagy markers, were not changed at the end of the ischemia or immediately after reperfusion. However, both the LC3-II and BECN1 levels were significantly decreased after 1, 2, or 4 h of reperfusion, and reached minimum values at 4 h (Fig. 1a). Thus, we selected 4 h of reperfusion to investigate whether the changes occurring during this phase were indicative of impaired autophagic flux via administration of the lysosomal inhibitor chloroquine (CQ). As shown in Fig. 1b, CQ alone increased the LC3-II/β-actin ratio in the sham group; conversely, the combination of I/R and the CQ treatment resulted in less LC3-II accumulation. In addition, SQSTM1-p62, a selective substrate of autophagy, was accumulated after reperfusion and remained increased in the I/R group treated with CQ (Fig. 1b). These observations were validated by morphological examination using transmission electron microscopy and immuno-electron microscopy. Intestinal tissues examined at 4 h after reperfusion showed fewer autophagic vacuoles (AVs) and reduced immunogold labeling than the sham group (Fig. 1c, Fig. S1A-B). These results demonstrate that autophagic flux was impaired during intestinal I/R.

To evaluate the role of autophagy during intestinal I/R, mice were treated with CQ prior to I/R. As shown in Table 1, serum IL-6 and TNF-α at 4 h after reperfusion were further increased by the administration of CQ. Intestinal fatty acid-binding protein (I-FABP), a sensitive marker of intestinal mucosal damage, showed the same trend. In addition, pretreatment with CQ worsened the mucosal damage and neutrophil infiltration in the I/R intestine (Fig. 1d). These data suggest that impaired autophagy during I/R contributes to intestinal injury.

**Table 1:** Effects of CQ on serum IL-6, TNF-α, and I-FABP levels during intestinal I/R

Group	IL-6 (pg/ml)	TNF-α (pg/ml)	I-FABP(ng/ml)
Sham	48.30 ± 5.14	149.04 ± 8.79	421.91 ± 62.15
Sham+CQ	59.64 ± 5.88	173.73 ± 9.71	465.32 ± 64.16
I/R	129.77 ± 9.02^a^	308.95 ± 16.65^a^	773.03 ± 93.38^a^
I/R+CQ	206.27 ± 14.75^b^	419.08 ± 13.79^b^	928.54 ± 72.73^b^

Results are presented as mean ± SD of 10 animals per group

^a^Significantly different (*P* < 0.01) from Sham

^b^Significantly different (*P* < 0.01) from I/R

### Identification of autophagy-related miRNAs that are dysregulated in response to intestinal I/R injury

**Fig. 2:**
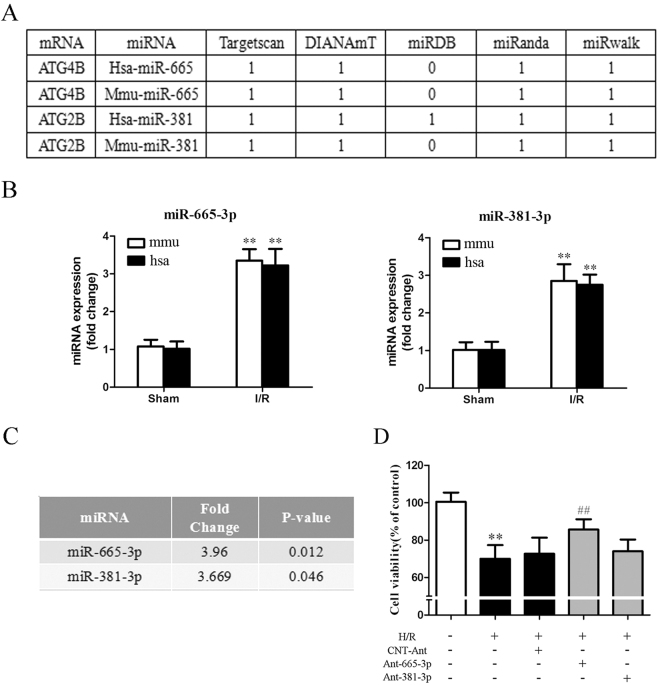
Identification of autophagy-related miRNAs in intestinal I/R. **a** ATG4B and ATG2B were predicted to be potential targets of miR-665-3p and miR-381-3p, respectively, in both humans and mice by different algorithms. **b** The expression of mature miRNAs under I/R conditions in mice and H/R conditions in Caco-2 cells (from humans) was quantified by qPCR. **c** Fold changes and *P* values of miR-665-3p and miR-381-3p expression in the microarray analysis. **d** Caco-2 cells were transfected with Ant-665-3p, Ant-381-3p or the negative control, as indicated, before being subjected to H/R. Cell viability was then determined using the CCK-8 assay, *n* = 6. ^**^P *<* 0.01 versus sham. ^##^*P* *<* 0.01 versus H/R

MiRNAs might play an important role in regulating autophagy by directly targeting ATGs^26,29^. We first performed a miRNA microarray analysis in murine intestine after I/R. We found that 131 of 1177 probe-miRNAs were differentially expressed in the I/R intestine (including 57 upregulated and 74 downregulated miRNAs, GEO no. GSE83701) compared with the sham group. We next focused on the 57 upregulated miRNAs, which might repress the autophagy activity (Fig. S2). An in silico analysis of ATGs was conducted to explore the potential roles of these miRNAs in autophagy during intestinal I/R (Table S1). Among these miRNAs, it was worth noting that miR-665-3p and miR-381-3p were predicted to target ATG4B and ATG2B, respectively, by at least four algorithms in both mice and humans (Fig. 2a). The results were further validated in vivo and in vitro by the TaqMan qRT-PCR assay and northern blotting (Fig. 2b, S3A). Moreover, our microarray analysis showed that miR-665-3p had a higher fold change and lower *P* value (Fig. 2c). Hypoxia/reoxygenation (H/R) of Caco-2 cells is a widely used in vitro model to mimic intestinal I/R in vivo^6^. Furthermore, we found that only miR-665-3p inhibition conferred protection against H/R injury in vitro (Fig. 2d).

Next, the molecular mechanism underlying the H/R-induced dysregulation of hsa-miR-665-3p was investigated. NF-κB signaling plays an important role in inflammation and apoptosis^5^, thus exploring the possible mechanisms between NF-κB and miR-665-3p is necessary. Furthermore, our results showed that H/R treatment induced NF-κB activation in Caco-2 cells, which was blocked by PDTC (a NF-κB inhibitor) treatment (Fig. S3B). PDTC treatment suppressed H/R-induced hsa-miR-665-3p upregulation and ATG4B downregulation (Fig. S3C-D). ChIP analysis further confirmed that NF-κB binds to the putative binding sites located at 3239–3249 upstream of the transcriptional start sites of hsa-miR-665-3p genes (Fig. S3E-F).

### MiR-665-3p negatively regulates ATG4B expression

**Fig. 3:**
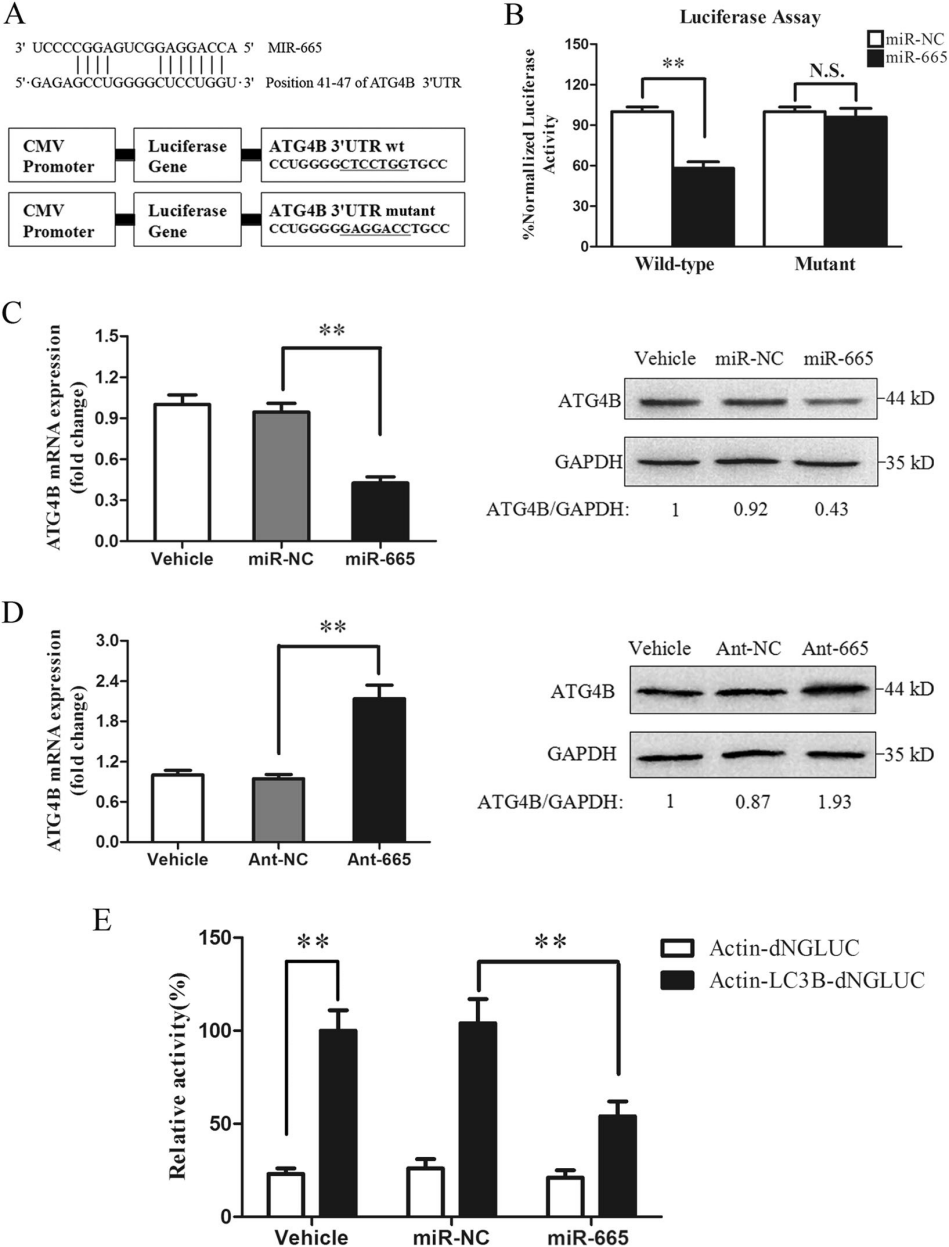
MiR-665-3p regulates ATG4B expression in Caco-2 cells. **a** A schematic presenting the luciferase constructs with the wild-type (wt) or mutant 3′UTR miR-665 MRE sequence of ATG4B. Mutations are underlined. **b** Normalized luciferase activity in lysates from Caco-2 cells that were co-transfected with the wild-type or mutant ATG4B-luciferase constructs and miR-665-3p or miR-NC, as indicated (*n* = 3). ^**^P *<* 0.01. N.S., not significant. **c**, **d** qPCR and western blot of ATG4B in cells that were treated with agomiR-665-3p or antagomiR-665-3p, *n* = 6. ^**^P *<* 0.01. **e** Normalized luciferase activity in culture medium from Caco-2 cells co-transfected with plasmids encoding Actin-dNGLUC or Actin-LC3B-dNGLUC together with CMV-Luc2 and miR-NC or miR-665-3p, *n* = 3. ^**^P *<* 0.01

We next investigated whether miR-665-3p directly targeted ATG4B mRNA. First, using TargetScan, we discovered the predicted interaction between miR-665-3p and ATG4B mRNA (Fig. S4A). Then, the region containing a potential miR-665-3p response element was cloned downstream of a luciferase reporter and expressed in Caco-2 cells (Fig. 3a). In the presence of miR-665-3p, the relative luciferase activity was repressed, indicating that miR-665-3p bound to the target 3′UTR of ATG4B mRNA. In contrast, miR-665-3p had no significant effect on the luciferase activity expressed from the mutant construct (Fig. 3b).

Importantly, overexpression of miR-665-3p inhibited ATG4B expression at the mRNA and protein levels (Fig. 3c). While transfection of cells with the miR-665-3p-specific antagomir (Ant-665) increased ATG4B expression (Fig. 3d). In addition, we used a cellular ATG4B activity assay to quantify the intracellular proteolysis of LC3B. miR-665-3p inhibited ATG4B enzymatic activity as indicated by the reduced cleavage of the engineered substrate (Fig. 3e). Similar results were observed in IEC-6 cells (Fig. S4B-D), indicating that the effects were not cell type-specific. These data demonstrate that miR-665-3p directly regulates ATG4B mRNA and protein levels as well as enzymatic activity by targeting its 3′UTR region.

### MiR-665-3p is involved in the regulation of ATG4B expression in human intestinal mesenteric infarction

**Fig. 4:**
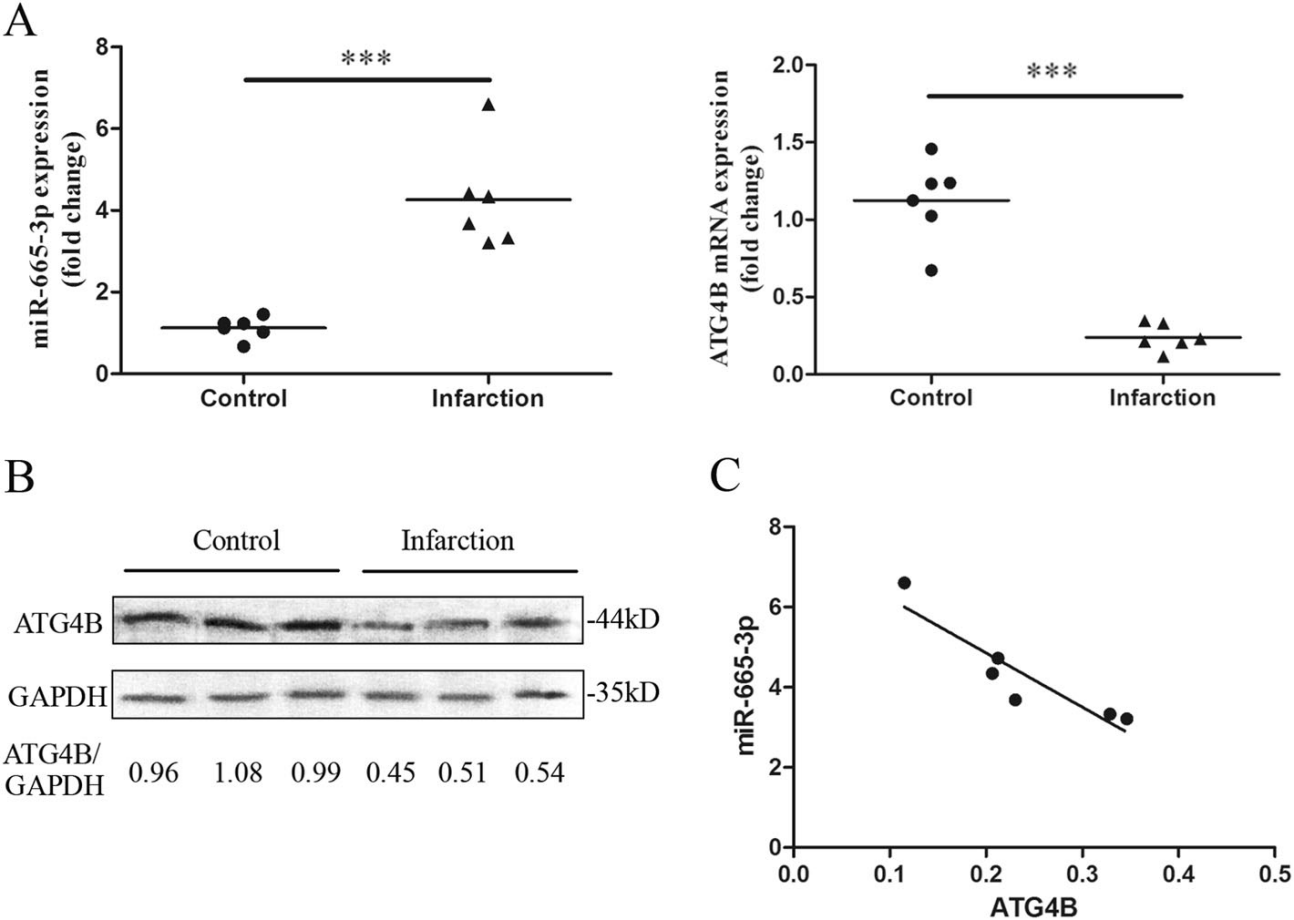
MiR-665-3p is involved in the regulation of ATG4B expression in human intestinal infarction. **a** Expression of miR-665-3p and ATG4B mRNA in tissue sections of human small intestine obtained from different areas of patients suffering from mesenteric infarction. ^***^P *<* 0.001. **b** Representative immunoblots of ATG4B expression in the control and intestinal infarction samples. **c** Inverse expression profile of miR-665-3p and ATG4B levels in the mucosa of the intestinal infarction samples (*n* = 6)

We next assessed whether miR-665-3p and ATG4B expression was dysfunctional in the terminal ileum mucosa from patients suffering from intestinal infarction. qRT-PCR analysis showed increased miR-665-3p expression and decreased ATG4B mRNA expression in the intestinal infarction biopsies compared to the normal intestinal tissues (Fig. 4a). We also showed that ischemic intestinal mucosa exhibited lower ATG4B protein levels (Fig. 4b). The miR-665-3p level was inversely correlated with the level of ATG4B in the intestinal infarction samples (Spearman *R*s = −0.9429, *P* *<* 0.05) (Fig. 4c), which indicated that the upregulation of miR-665-3p could be a mechanism underlying the downregulation of ATG4B in infarcted human intestinal tissues.

### Overexpression of miR-665-3p suppresses autophagic activity and aggravates H/R injury in vitro

**Fig. 5:**
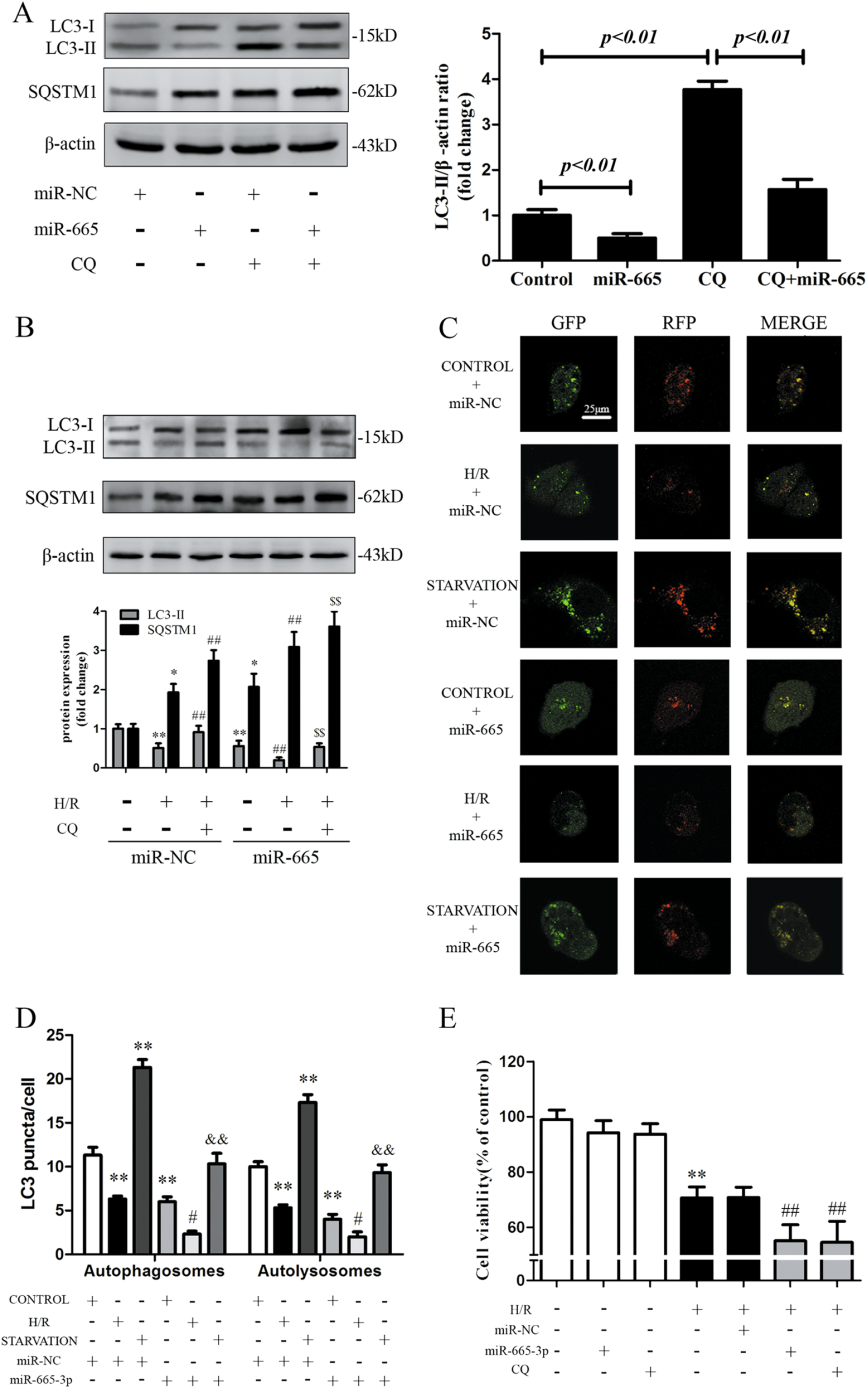
Overexpression of miR-665-3p suppresses autophagic activity and aggravated H/R injury in Caco-2 cells. **a** Caco-2 cells were transfected with either miR-NC or miR-665-3p in the absence or presence of CQ (20 μM). Whole-cell extracts were subjected to immunoblotting with anti-LC3 and anti-SQSTM1 antibodies. **b** Representative immunoblots of endogenous LC3B-II and SQSTM1 expression in Caco-2 cells that were transfected with either miR-NC or miR-665-3p and subjected to H/R and CQ treatment (*n* = 3). **c** Representative images of LC3 staining in different groups of Caco-2 cells that were infected with mRFP-GFP-LC3 adenovirus for 24 h. **d** Quantitative analysis of autophagosome and autolysosome formation (*n* = 3). **e** The cells were incubated with 20 μM CQ for 6 h or transfected with either miR-NC or miR-665-3p before H/R was induced. Then, cell viability was assessed using the CCK-8 assay (*n* = 8). ^*^P *<* 0.05 versus control, ^**^P *<* 0.01 versus control, ^#^*P* *<* 0.05 versus H/R, ^##^*P* *<* 0.01 versus H/R, ^$$^*P* *<* 0.01 versus H/R+CQ, ^&&^*P* *<* 0.01 versus starvation

Next, the involvement of miR-665-3p in autophagic flux and its role in response to H/R was examined. As shown in Fig. 5a, cotreatment with miR-665-3p and CQ resulted in reduced LC3-II accumulation compared with CQ treatment alone, which confirmed that miR-665-3p could inhibit autophagic flux in vitro. In addition, miR-665-3p overexpression further aggravated the H/R-induced reduction in LC3-II accumulation and SQSTM1-p62 augmentation in the presence or absence of CQ (Fig. 5b). We next utilized the mRFP-GFP-LC3 adenovirus construct to further explore autophagic flux. Autophagosomes show both green and red fluorescence, generating yellow puncta^30^. As shown in Fig. 5c, d, miR-665-3p decreased the yellow fluorescent signals to levels lower than those observed in control cells and cells subjected to H/R and starvation. To further confirm that miR-665-3p modulated autophagy through its effects on ATG4B, we performed rescue experiments by overexpressing ATG4B (Fig. S5A-C), which indicated the miR-665-3p-mediated autophagy suppression was reversed upon co-expression of the ATG4B protein

Then, we examined the effect of miR-665-3p on cell survival upon H/R. Cell viability analysis showed that the deleterious effects due to H/R treatment were aggravated by miR-665-3p overexpression, which was similar to the autophagy inhibitor CQ (Fig. 5e). Taken together, these data strongly suggest that the overexpression of miR-665-3p suppressed ATG4B-mediated autophagic flux and aggravated the H/R injury in Caco-2 cells.

### Inhibition of miR-665-3p stimulates autophagic flux and mitigates H/R injury in Caco-2 cells

**Fig. 6:**
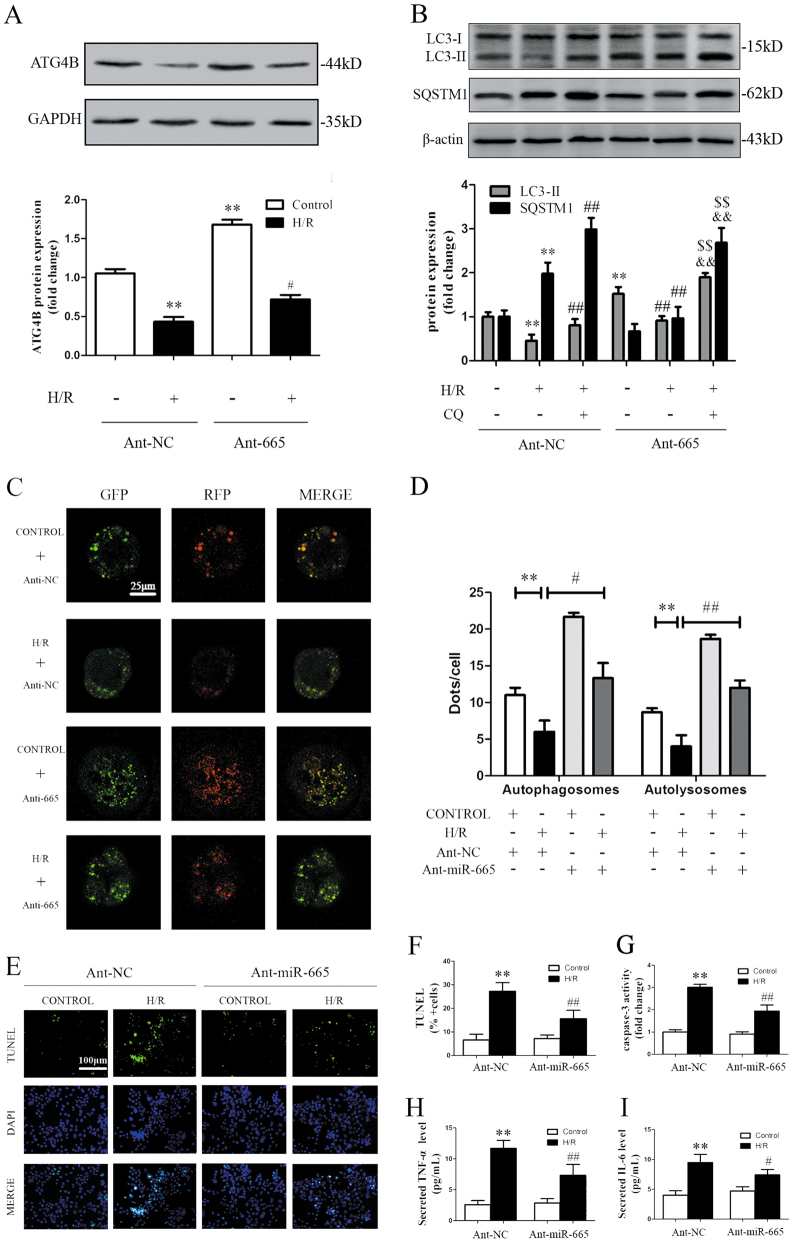
Inhibition of miR-665 stimulates autophagic flux and mitigates cell apoptosis and inflammation in Caco-2 cells treated with H/R. **a** Ant-665 stimulated ATG4B expression in response to H/R. **b** Representative immunoblots of endogenous LC3B-II and SQSTM1 in Caco-2 cells that were transfected with Ant-NC or Ant-665 and subjected to H/R (*n* = 3). **c** Representative images showing LC3 staining in different groups of Caco-2 cells that were infected with the mRFP-GFP-LC3 adenovirus for 24 h. **d** Quantitative analysis of autophagosome and autolysosome formation (*n* = 3). **e** TUNEL and DAPI staining. **f** The percentage of apoptotic cells is represented as TUNEL-positive cells/DAPI (*n* = 6). **g** Caspase-3 activity (*n* = 6). **h**, **i** Secreted TNF-α and IL-6 in the cell culture supernatant were quantified by ELISA (*n* = 6). ^**^P *<* 0.01 versus the control, ^#^*P* *<* 0.05, ^##^*P* *<* 0.01 versus H/R, ^&&^*P* *<* 0.01 versus H/R+CQ, ^$$^*P* *<* 0.01 versus H/R + Ant-665

Next, we evaluated the effect of miR-665-3p inhibition on autophagy in Caco-2 cells under H/R injury. As shown in Fig. 6a, b, Ant-665 blocked the H/R-induced suppression of ATG4B and recovered the impaired autophagy. Under H/R conditions, treatment with Ant-665 in the presence of lysosomal inhibition (CQ) further increased the LC3-II levels compared with CQ alone and Ant-665 alone. Fluorescent imaging of mRFP-GFP-LC3 also showed that Ant-665 considerably increased the number of yellow (autophagosomes) LC3 puncta under non-ischemic or H/R conditions (Fig. 6c, d). The above results suggest that miR-665-3p inhibition stimulated autophagic flux in response to H/R.

Cell apoptosis and inflammatory responses contribute to tissue damage in response to H/R^31,32^. As shown in Fig. 6e, f, the number of TUNEL-positive cells was significantly increased after H/R but was reduced after the application of Ant-665. Meanwhile, miR-665-3p inhibition alleviated caspase-3 activity (Fig. 6g). In addition, the levels of pro-inflammatory cytokines, such as TNF-α and IL-6, were reduced in the Ant-665 group (Fig. 6h, i). These results indicate that the inhibition of miR-665-3p protected Caco-2 cells from H/R by alleviating apoptosis and inflammation, which may be induced by the recovered autophagic flux.

### ATG4B is indispensable for the suppression of inflammation and apoptosis induced by miR-665-3p inhibition under H/R conditions

**Fig. 7:**
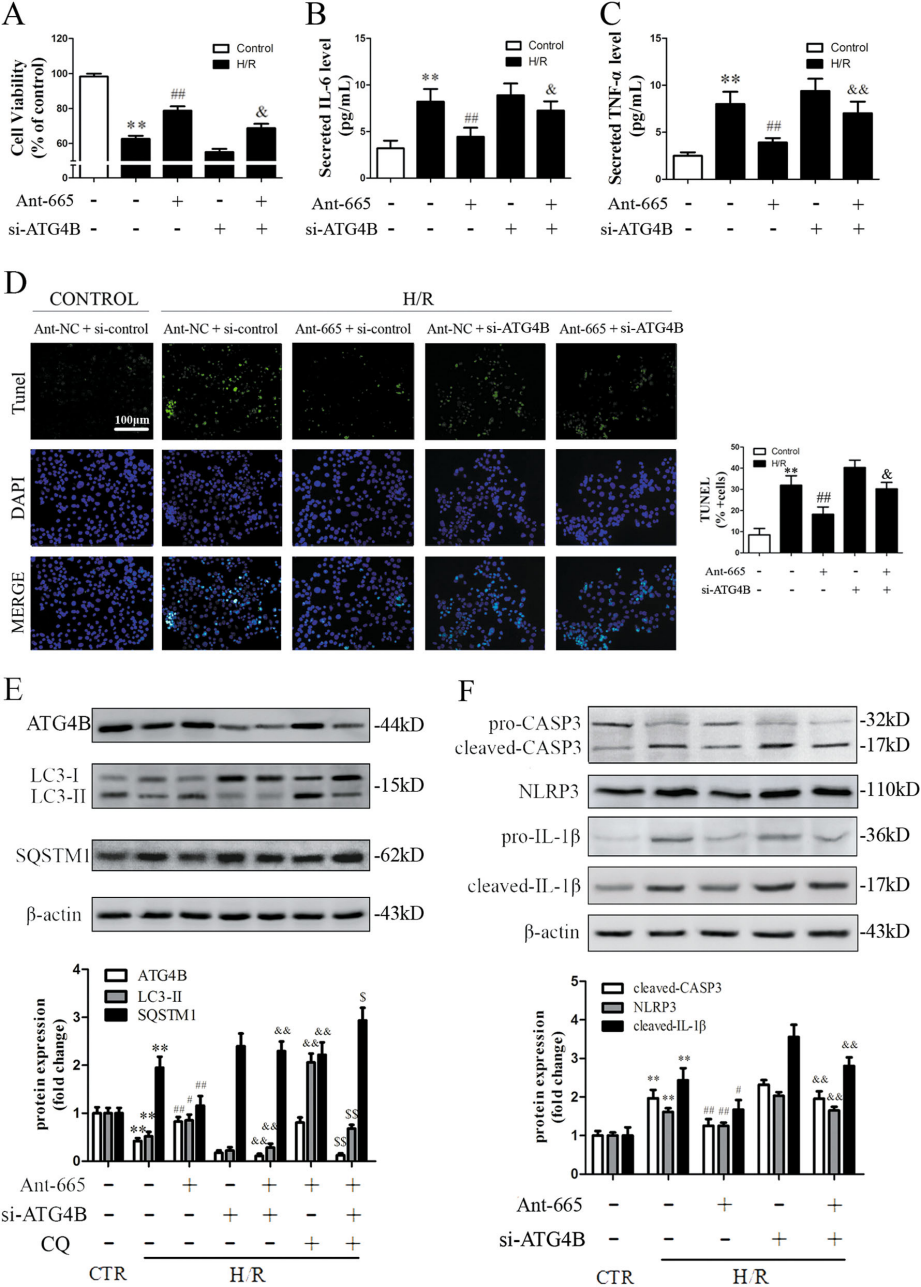
ATG4B is indispensable for the suppression of inflammation and apoptosis induced by miR-665 inhibition under H/R conditions. Caco-2 cells were co-transfected with Ant-665, si-ATG4B, Ant-NC and CQ as indicated. **a** Cell viability was assessed using the CCK-8 assay (*n* = 6). **b**, **c** Secreted TNF-α and IL-6 in the cell culture supernatant were quantified by ELISA (*n* = 6). **d** TUNEL and DAPI staining. The percentage of apoptotic cells is represented as TUNEL-positive cells/DAPI (*n* = 6). **e** Representative immunoblots of endogenous ATG4B, LC3B-II, and SQSTM1 in different groups of intestinal tissues (*n* = 3). **f** Representative immunoblots for pro- and cleaved CASP3, NLRP3, and pro- and cleaved IL-1β in different groups (*n* = 3). ^**^P *<* 0.01 versus the control, ^#^*P* *<* 0.05, ^##^*P* *<* 0.01 versus H/R, ^&^*P* *<* 0.05, ^&&^*P* *<* 0.01 versus H/R + Ant-665, ^$^*P* *<* 0.05, ^$$^*P* *<* 0.01 versus H/R + Ant-665+CQ

We next determined whether ATG4B was responsible for the protective effects induced by miR-665-3p inhibition. As shown in Fig. 7a–d, Ant-665 improved cell viability, reduced apoptosis, and alleviated pro-inflammatory cytokine release under H/R conditions, as described above. However, it did not confer such protection after ATG4B knockdown, despite the downregulation of miR-665-3p. To further validate the potential mechanisms, we examined the autophagic activity of each group. As expected, Ant-665 enhanced ATG4B expression and recovered the autophagic activity under H/R conditions, but ATG4B knockdown significantly abolished this trend (Fig. 7e). In addition, Ant-665 attenuated caspase-3 cleavage, NLRP3 expression, and IL-1β maturation after H/R, while ATG4B knockdown mitigated these effects (Fig. 7f). Taken together, these data reveal that ATG4B is required for the regulation of Ant-665-mediated protection against H/R injury.

### Inhibition of miR-665-3p blocks I/R-induced downregulation of ATG4B expression, reinforces autophagic activity, and confers protection against intestinal I/R in vivo

**Fig. 8:**
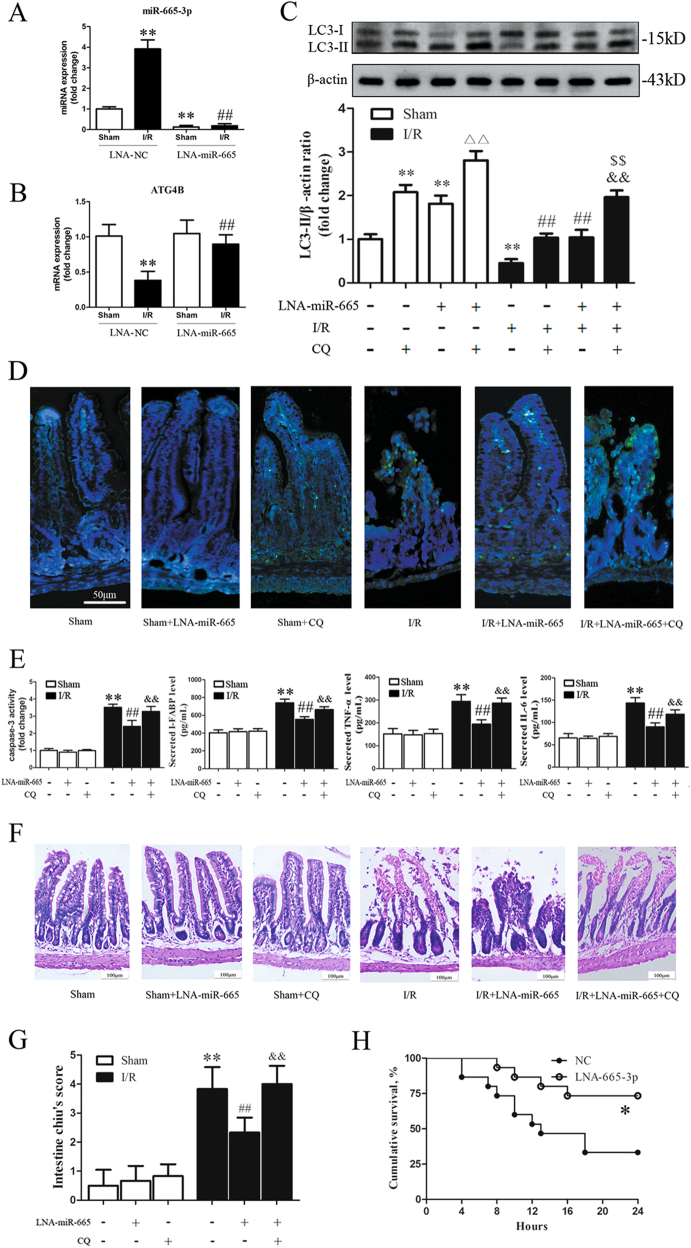
Inhibition of mmu-miR-665 blocks I/R-induced downregulation of ATG4B expression and reinforces autophagic activity, leading to decreased inflammation and apoptosis. **a**, **b** qPCR analysis of miR-665-3p and ATG4B in LNA-miR-665-3p-treated tissues (*n* = 6). **c** Representative immunoblots of endogenous LC3B-II in different groups of intestinal tissues (*n* = 6). **d** Intestinal tissue sections were labeled for the TUNEL assay. Apoptotic nuclei are stained green, and nuclei counterstained with DAPI are blue (*n* = 6). **e** Caspase-3 activity in the intestinal tissues and secreted I-FABP, TNF-α and IL-6 in serum were quantified by ELISA (*n* = 6). **f**, **g** Representative H&E staining and histological injury scores of the gut in the different groups (*n* = 6). ^**^P *<* 0.01 versus sham, ^##^*P* *<* 0.01 versus I/R, ^△△^*P* *<* 0.01 versus sham+CQ, ^&&^*P* *<* 0.01 versus I/R+CQ, ^$$^*P* *<* 0.01 versus I/R + LNA-miR-665. **h** Alterations in the survival rate of mice after intestinal I/R. Mice received LNA-NC or LNA-miR-665-3p through caudal vein injection 12 h before they were subjected to intestinal ischemia for 45 min, and the mice were observed for 24 h (*n* = 15). ^*^P *<* 0.05 versus LNA-NC

To examine the possibility that targeting miRNA in vivo can modulate the autophagy response and alleviate injury during intestinal I/R, we generated miR-665-3p-knockdown mice using locked nucleic acid-modified antisense oligonucleotides targeting miR-665-3p (LNA-665) (Fig. 8a). After intestinal I/R, pretreatment with LNA-665 prevented I/R-induced suppression of ATG4B expression compared to the negative control (NC) group (Fig. 8b). During either sham or I/R treatment, the LNA-665-induced accumulation of LC3-II was enhanced by co-treatment with CQ compared with either LNA-665 alone or CQ alone (Fig. 8c). Administration of LNA-665 alleviated I/R-induced apoptosis, inflammation, and histological injury, while these protective effects were suppressed after the administration of CQ (Fig. 8d–g). To evaluate the long-term protective effects of LNA-665 treatment, we conducted a 24-h survival study in mice after intestinal I/R. As Fig. 8h shows, the overall survival rate of the LNA-665-treated group was significantly higher than that in the NC-treated group. Collectively, our data show that miR-665-3p inhibition alleviated intestinal I/R injury in vivo and improved survival by inducing autophagy.

## Discussion

Impaired autophagy contributes to diverse disease processes by increasing systemic inflammation and cell apoptosis^7,33,34^, but its specific regulatory mechanisms in intestinal I/R are elusive. Several researchers have reported impaired autophagy and miRNA dysfunction in I/R injury^28,35^. However, the regulation of autophagy by miRNAs and their therapeutic potential in intestinal I/R have not been thoroughly addressed. To the best of our knowledge, this study presents the first evidence of the following four findings. (1) Autophagic flux is impaired during the early reperfusion stage, playing a crucial role in intestinal I/R injury. (2) Based on microarray and conservation analyses, miR-665-3p is significantly upregulated among the miRNAs targeting ATGs in the intestinal I/R mouse model. (3) Inhibition of miR-665-3p recovers the cytoprotective autophagy to alleviate inflammation and apoptosis in response to I/R in vivo and in vitro. (4) ATG4B plays a critical role in miR-665-3p inhibition-mediated protection during I/R. The present study describes new insights into the implications of autophagy-targeting miRNAs in intestinal I/R.

Some researchers have recently explored the effect of autophagy on intestinal I/R^36,37^. However, the involvement of autophagy at different phases during reperfusion remained elusive. In this study, we first explored the changes in intestinal autophagic activity during ischemia followed by 0–8 h of reperfusion. Interestingly, the basic level of intestinal autophagy decreased gradually during the early reperfusion period (0–4 h) and reached the lowest level at 4 h. The further results indicate that impaired autophagy is responsible for I/R-induced intestinal damage. In accordance with our research, Yun and Ma demonstrated that the impaired autophagy during liver I/R or cardiac I/R is inadequate to prevent cell death and contributes to the reperfusion injury^12,13^. However, autophagy is potentiated during reperfusion after cerebral ischemia, and it plays different roles in permanent and transient artery occlusion^38^. It is possible that the effects of reperfusion on autophagic flux vary with the extent and severity of ischemia, due to the tolerance of different organs to ischemia. These findings prompted us to further explore the molecular mechanism by which autophagy impairment is regulated during intestinal I/R.

An increasing number of miRNAs are found to be key endogenous regulators of multiple biological processes, including cell apoptosis, differentiation, proliferation, metabolism and inflammation^39^. Zhu et al.^40^ showed that miR-30a modulated autophagic activity by inhibiting BECN1 (ATG6) expression, which is the first report on the roles of miRNAs in regulating autophagy. Here, we explored the miRNA expression profiles associated with impaired autophagy during intestinal I/R in mice. Among the 57 upregulated miRNAs, we chose miR-665-3p for further exploration because it is a highly conserved ATG-targeting miRNA and its protective effect on Caco-2 cells during H/R (Table S1, Fig. 2d). Next, our gain- and loss-of-function analyses showed that miR-665-3p repressed ATG4B expression. Collectively, these data suggest that miR-665-3p is a new autophagy regulator that targets ATG4B in mice and humans, suggesting it may affect intestinal I/R injury.

Accumulating evidence suggests the potential protective roles of specific miRNAs in the pathogenesis of I/R injury. Silencing miR-24 or miR-92a ameliorated renal or cardiac I/R injury, respectively^27,28^. Here we provide evidence of the relevance of miR-665-3p in intestinal I/R or H/R. As expected, we discovered that miR-665-3p overexpression inhibited autophagic flux, and it aggravated H/R injury in Caco-2 cells. In contrast, miR-665-3p inhibition significantly increased autophagic flux and decreased the number of TUNEL-positive cells and the levels of pro-inflammatory factors during H/R. These data were validated by miR-665-3p knockdown in mice suffering from intestinal I/R.

Besides influencing apoptosis and inflammation via autophagy regulation, a recent study showed that upregulation of miR-665 promotes apoptosis and colitis in inflammatory bowel disease by repressing the endoplasmic reticulum (ER) stress components XBP1 and ORMDL3^41^. We obtained similar findings in our model. miR-665-3p inhibition restored XBP1 and ORMDL3 expression under I/R and relieved H/R via XBP1 (Fig. S6A-F), suggesting that inhibition of miR-665-3p may also attenuate intestinal I/R via ER stress.

The autophagic process involves a group of ATG genes that are conserved from yeast to humans. ATG-mediated autophagy plays a crucial role in the control of inflammatory and apoptotic signaling^42^. Paneth cells from ATG16L1 hypomorphic mice show enhanced transcription of pro-inflammatory cytokines and adipokines^43^. ATG7-deficient macrophages activate inflammasomes, leading to higher levels of IL-1β in response to LPS^44^. As a novel cysteine protease, ATG4B exerts its function as both a processing and a deconjugating protease in the efficient progression of autophagy^45^. Notably, ATG4B plays a crucial role in intestinal homeostasis, as evidenced by the increased susceptibility of ATG4B-deficient mice to develop colitis^16^. In our study, enforced expression of ATG4B by miR-665-3p inhibition stimulated autophagic activity, reduced the levels of TNF-α and IL-6, inhibited NLRP3 expression and IL-1β maturation and alleviated caspase-3 activation. In fact, it has been shown that macrophages lacking autophagy do not efficiently degrade their engulfed cargo and produce increased amounts of pro-inflammatory cytokines^46^. Autophagy deficiency in myeloid cells causes aberrant activation of the inflammasome, leading to the development of inflammatory disorders^47^. Thus, we propose that the increased autophagic activity after miR-665-3p inhibition may contribute to the alleviation of the inflammatory responses induced by intestinal I/R. A single miRNA can target multiple mRNAs. Although ATG7 and ATG16L1 were also predicted as target genes of miR-665-3p (Table S1), ATG7 is dispensable for gut homeostasis, and ATG16L1 causes irreversible impairment of autophagy^44,48^. ATG4B knockdown abolished the resistance of Ant-665 to inflammation activation and apoptosis induction under H/R conditions. Therefore, the Ant-665-induced protection against H/R injury is ATG4B-dependent.

Locked nucleic acid (LNA)-modified oligonucleotides represent a new frontier in miRNA silencing with high binding efficiencies, improved stability, and excellent tissue uptake^49^. Numerous clinical trials have focused on the therapeutic silencing of disease-related miRNAs using the LNA-modified-anti-miR oligonucleotide approach in vivo to treat multiple disorders^50,51^. The LNA-modified miR-122 inhibitor miravirsen was recently shown to successfully induce a dose-dependent and prolonged decrease in the HCV RNA levels of patients with chronic HCV genotype 1 infection (phase 2 clinical trials) without evidence of viral resistance^52^. Our previous research demonstrated that LNA-miR-34a alleviated intestinal I/R injury through SIRT1-mediated ROS accumulation and apoptosis^6^. In this study, we found that LNA-miR-665 reduced the miR-665-3p level, enhanced ATG4B expression and recovered the basic autophagic activity. LNA-miR-665 also significantly attenuated the histological injury and improved the survival rate. These results show the feasibility of targeting miRNAs with LNA to modulate autophagic activity and alleviate intestinal I/R injury in vivo.

Apart from autophagy, previous studies also showed that programmed necrotic cell death and pyroptosis of enterocytes played important roles after intestinal injury^53,54^. Suppression of autophagic flux contributed to RIP1-RIP3-dependent necroptosis and Pu reported that loss of Atg7 resulted in increased production of IL-1β and pyroptosis^55,56^. Based on the above research and our findings, it seems that impaired autophagy under intestinal I/R increases apoptosis, and may also increase necroptosis and pyroptosis. The parallel existence of multiple types of cell death demonstrates the complexity of the pathophysiology of intestinal I/R. The detailed mechanisms and regulatory processes require further exploration.

In conclusion, the current findings are the first to demonstrate that targeting the miR-665-3p-ATG4B-autophagy pathway alleviates systemic inflammatory responses and excessive apoptosis induced by intestinal I/R injury. Our study may contribute to the improvement of intestinal I/R therapeutics through treatment with LNA-miR-665. This study may lead to rational target selection for therapeutic intervention in patients suffering from gut ischemic diseases.

## Materials and methods

### Animals and treatment

Male C57BL/6 mice (aged 8 weeks) weighing 18–22 g were obtained from the Animal Center of Dalian Medical University and housed under standard laboratory conditions with access to standard laboratory chow and water. The murine intestinal I/R model was established as described previously^6^. Briefly, the superior mesenteric artery was occluded with a microvascular clamp for 45 min, and then the clamp was removed for reperfusion. Normal saline and chloroquine (CQ; Sigma, C6628; 60 mg/kg) were given intraperitoneally 30 min prior to ischemia in accordance with a previous study^12^. All procedures were conducted according to the Institutional Animal Care Guidelines and were approved by the Institutional Ethics Committee of Dalian Medical University.

### Cell culture, H/R procedures, and cell viability determination

Caco-2 cells (ATCC, HTB-37) and IEC-6 cells (ATCC, CRL-1592) were cultured in DMEM (Gibco, 11965), supplemented with 10% fetal bovine serum (Gibco, 10099–141), 1% non-essential amino acids (Sigma, M7145), and 1% glutamine (Sigma, G3126) at 37 °C in a humidified atmosphere of 5% CO_2_. The identity of these cell lines (Caco-2 and IEC-6 cells) was authenticated with STR profiling. The cells were starved in Earle’s Balanced Salt Solution (EBSS, Sigma, E7510) to induce autophagy. The cells were incubated in a microaerophilic system (Thermo, Waltham, MA) with 5% CO_2_ and 1% O_2_ balanced with 94% N_2_ for 12 h to simulate hypoxic conditions. The cells were then cultured under normoxic conditions for 6 h to achieve reoxygenation.

Cell viability was evaluated using a Cell Counting Kit-8 (CCK-8, Dojindo, CK04), according to the manufacturer’s instructions. Briefly, the cells were plated on 96-well plates. After H/R, CCK8 solution was added to each well and the cells were incubated at 37°C for 3 h. Optical densities were measured at 450 nm using a microplate reader (Biotec, USA).

### Human tissue samples

The study was approved by the Institutional Ethics Committee of Dalian Medical University (Dalian, China), and the samples were collected after informed written consent was obtained. A total of six samples were collected from six patients who underwent an operation for acute mesenteric arterial embolism, strangulated intestinal obstruction, or incarcerated hernia (Supplementary Table S2). The tissue samples were immediately snap-frozen for western blot and qRT-PCR.

### Morphology

Intestines were removed and fixed by inflation with 4% paraformaldehyde in 1 × PBS at continuous pressure of 25 cm H_2_O and were embedded in paraffin. Sections were stained with H&E and scored blindly for the severity and extent of intestinal lesions according to Chiu’s score^57^.

### Transmission electron microscopy

Four hours after reperfusion, the mice were euthanized with choral hydrate and perfused with precooled PBS (pH 7.4), followed by PBS containing 4% paraformaldehyde and 0.25% glutaraldehyde. The intestines were then removed and incubated overnight in 2% paraformaldehyde and 2.5% glutaraldehyde in 0.1 M PBS (pH 7.4). The target tissues were cut into 50-µm-thick sections using a vibratome. Selected areas of intestines were processed by postfixation in 1% osmium tetroxide for 1 h, dehydrated in a graded ethanol series, and embedded in epoxy resin. Polymerization was performed at 80 °C for 24 h. Ultrathin sections (100 nm) were cut, stained with uranyl acetate and lead citrate, and viewed under a transmission electron microscope (JEOL, JEM2000EX, Japan). We captured five nonrepeating micrographs for each sample in randomly selected fields, and the area of one such micrograph was regarded as the unit area. The numbers of autophagic vacuoles (AVs) per unit area in each sample were counted and analyzed.

To further determine the properties of the autophagosome-like vesicles, immuno-electron microscopy was performed as previously described^58,59^. Briefly, ultrathin sections were incubated with antibodies targeting LC3B (1:100) followed by treatment with 10-nm Colloidal Gold-AffiniPure goat anti-rabbit IgG (1:40). Finally, the gold labeling was measured using a transmission electron microscope.

### Enzyme-linked immunosorbent assay (ELISA)

The amounts of I-FABP, IL-6, and TNF-α secreted in the mouse serum or supernatants from Caco-2 cells cultured in DMEM containing penicillin*/*streptomycin in an atmosphere containing 5% CO_2_ at 37°C were determined by ELISA (R&D Systems), according to the manufacturer’s instructions.

### MiRNA microarray analysis

The microarray analysis for miRNA profiling was conducted by Shanghai Kangcheng Technology using the miRCURY LNA Array system (Exiqon, Vedbaek, Denmark). Total RNA was isolated using TRIzol (Invitrogen) and purified with an RNeasy Mini Kit (QIAGEN), according to the manufacturer’s instructions. RNA quality and quantity were measured using a NanoDrop spectrophotometer (ND-1000, NanoDrop Technologies), and RNA integrity was determined by gel electrophoresis. After quality control, the miRCURY™ Hy3™/Hy5™ Power labeling kit (Exiqon, Vedbaek, Denmark) was used to label the miRNAs, according to the manufacturer’s guidelines. After stopping the labeling procedure, the Hy3™-labeled samples were hybridized on the miRCURY^TM^ LNA Array (v.18.0) (Exiqon), according to the array manual. Then, the slides were scanned using the Axon GenePix 4000B microarray scanner (Axon Instruments, Foster City, CA). The raw data were normalized using the quantile algorithm. The threshold value for significance that was used to define the upregulated miRNAs was a fold change ≥ 2.0 and a *P* *≤* 0.05. The microarray data discussed in this paper have been deposited in the NCBI Gene Expression Omnibus and are accessible through GEO Series accession number GSE83701.

### MiRNA target prediction

The potential target genes of miRNAs were predicted in silico using different miRNA target prediction algorithms: TargetScan (http://www.targetscan.org/), DIANA-MICROT (http://diana.cslab.ece.ntua.gr/micro-CDS/), miRDB (http://mirdb.org/miRDB/), miRanda (http://www.microrna.org/microrna/), and miRwalk (http://zmf.umm.uni-heidelberg.de/apps/zmf/mirwalk2/).

### Dual luciferase-reporter assay

Vectors containing wild-type or mutant miR-665-3p MREs from the ATG4B 3′UTR were obtained from GenePharma (Shanghai, China) and cotransfected with miR-665-3p into Caco-2 cells. Forty-eight hours later, the cells were lysed. Firefly and Renilla luciferase activities were measured with a Dual-Luciferase Reporter Assay Kit (TransGen, FR201-02) using a dual luciferase-reporter assay system (Berthold, Germany). The results were evaluated by normalizing the firefly luciferase activity to the Renilla luciferase activity.

### Transfection of agomirs, antagomirs, plasmid, and siRNA

Agomirs, antagomirs, the pcDNA3.1-ATG4B plasmid, si-ATG4B, si-XBP1, and the negative control (sequences are listed in Supplementary Table 3) were obtained from GenePharma (Shanghai, China). Cell transfection and co-transfection experiments were performed with nucleic acids using Lipofectamine 3000 (Life Technologies, L3000015), according to the manufacturer’s protocols.

### Generation of a miR-665-3p-knockdown mouse model

Knockdown (KO) mice were constructed as previously described^6^. Briefly, LNA-665 (Exiqon, Vedbaek, Denmark) and the corresponding control (LNA-NC) were diluted in saline and administered by caudal vein injection at a dose of 2 mg/kg 12 h before surgery. All sequences are listed in Supplementary Table 3.

### **mRFP-GFP-LC3 adenoviral vector**

Caco-2 cells were plated in a six-well plate and allowed to reach 50–70% confluence by the time of transfection. mRFP-GFP-LC3 adenoviral vectors were purchased from HanBio Technology Co., Ltd. (HanBio, HB-LP210 0001). Adenoviral infection was performed according to the manufacturer’s instructions. Caco-2 cells were incubated in growth medium with the adenoviruses at an MOI of 50 for 24 h at 37 °C and were then grown in medium under vehicle, starvation, or H/R conditions for the appropriate times at 37 °C. Autophagy was observed under a TCS SP5 laser scanning confocal microscope (Leica, Wetzlar, Germany). Autophagic flux was determined by evaluating the numbers of GFP and mRFP puncta (the numbers of puncta/cell were counted).

### RNA extraction and quantitative RT-PCR (qRT-PCR)

MiRNAs were extracted using an miRcute miRNA isolation kit (TIANGEN Biotechnology, DP501). The mature miRNAs were quantified using TaqMan Hairpin-it miRNA and a U6 snRNA normalization quantitation kit (GenePharma, E01008). Total RNAs were extracted using RNAiso Plus reagent (TAKARA, 9108). ATG4B mRNA expression was quantified using a SYBR Green Quantitative RT-PCR kit (TransGen, AQ101-03). Changes in the miRNA or mRNA levels were quantified by the 2^−ΔΔCT^ method using U6 or β-actin mRNA as control. The primers used during the study were as follows: ATG4B primers 5′-GGCAAGTCTATAGGCCAGTGGTA-3′ and 5′-GGCCCTGCATAACCTTCTGA-3′; and β-actin primers 5′-ACTGCCGCATCCTCTTCCT-3′ and 5′-TCAACGTCACACTTCATGATGGA-3′. The reactions were performed in duplicate, and the number of independent experiments was marked.

### Immunoblotting

Total proteins were extracted from intestinal tissues with a total protein extraction kit (Beyotime, P0013), and the cells were lysed with RIPA buffer. Protein concentration was quantified using a BCA protein assay kit (Beyotime, P0011). A total of 25 μg protein was loaded on either 10% or 15% SDS-polyacrylamide gels. After electrophoresis, the gels were electrotransferred onto PVDF membranes and then the membranes were blocked with 5% non-fat-dried milk in PBS containing 0.1% Tween-20. The membranes were then probed with the following relevant primary antibodies: anti-BECN1 ab (Cell Signaling Technology, 3495), anti-LC3B ab (Cell Signaling Technology, 2775), anti-SQSTM1/p62 ab (Proteintech Group, 18420-1-AP), anti-ATG4B ab (Abcam, ab154843), anti-IκB-α ab (Abcam, ab32518), anti-β-actin ab (ZSGB-BIO, TA-09), anti-pro/active-CASP3/caspase-3 ab (Abcam, ab13585), anti-NLRP3 ab (Cell Signaling Technology, 15101), anti-IL-1β ab (R&D Systems, Inc., MAB601), anti-XBP1 ab (Abcam, ab37152), anti-ORMDL3 ab (Abcam, ab211522), anti-α-tubulin ab (Abcam, ab7291) and anti-GAPDH ab (Abcam, ab8245). After three washes with PBT, the membranes were incubated with the corresponding secondary antibodies. The membranes were then exposed to enhanced chemiluminescence-plus reagents (Advansta Inc., K-12043). The emitted light was captured by a BioSpectrum 410 multispectral imaging system with a Chemi 410 h camera and analyzed with Gel-Pro Analyzer Version 4.0 (Media Cybernetics, MD, USA).

### TUNEL and caspase-3 activity assays

Paraffin-embedded intestine sections were deparaffinized in xylene and rehydrated with decreasing concentrations of ethanol. Apoptosis was detected by in situ TUNEL (terminal deoxynucleotidyltransferase biotin-dUTP nick end labeling) using an apoptosis detection kit (Roche, 11684817910) according to the manufacturer’s instructions. The nuclei were stained with DAPI (Beyotime, C1005). Caspase-3 activity in the cell lysates was measured using a caspase-3 activity assay kit (Beyotime, C1005), according to the manufacturer’s protocol.

### Statistics

All experimental data are reported as the mean ± SD. Statistical analyses were performed using two-tailed Student’s *t*-test, and differences in survival between groups were analyzed using the Kaplan–Meier log-rank test. In experiments with more than two groups, normally distributed data were analyzed by multifactorial one-way analysis of variance (ANOVA) followed by Tukey’s post hoc test. Non-normally distributed data were compared using the Kruskal–Wallis test followed by the Wilcoxon rank sum test with Bonferroni adjustments. All statistical analyses were performed using GraphPad Prism 5.0 (GraphPad Prism Software, La Jolla, CA, USA). *P*-values less than 0.05 were considered statistically significant.

## Electronic supplementary material

Supplementary Materials and Methods

Supplementary Figure 1

Supplementary Figure 2-6&Tables

## References

[1] Matsuda A (2014). FK866, a visfatin inhibitor, protects against acute lung injury after intestinal ischemia-reperfusion in mice via NF-κB pathway. Ann. Surg..

[2] Eltzschig HK, Eckle T (2011). Ischemia and reperfusion—from mechanism to translation. Nat. Med..

[3] Wu MCL (2013). The receptor for complement component C3a mediates protection from intestinal ischemia-reperfusion injuries by inhibiting neutrophil mobilization. Proc. Natl Acad. Sci. USA.

[4] Grootjans J (2011). Level of activation of the unfolded protein response correlates with paneth cell apoptosis in human small intestine exposed to ischemia/reperfusion. Gastroenterology.

[5] Chen LW (2003). The two faces of IKK and NF-kappaB inhibition: prevention of systemic inflammation but increased local injury following intestinal ischemia-reperfusion. Nat. Med..

[6] Wang G (2016). miR-34a-5p inhibition alleviates intestinal ischemia/reperfusion-induced reactive oxygen species accumulation and apoptosis via activation of SIRT1 signaling. Antioxid. Redox Signal..

[7] Andrade-Oliveira V (2015). Gut bacteria products prevent AKI induced by ischemia-reperfusion. J. Am. Soc. Nephrol..

[8] Klionsky DJ (2016). Guidelines for the use and interpretation of assays for monitoring autophagy (3rd edition). Autophagy.

[9] Galluzzi L, Pietrocola F, Levine B, Kroemer G (2014). Metabolic control of autophagy. Cell.

[10] Choi AMK, Ryter SW, Levine B (2013). Autophagy in human health and disease. N. Engl. J. Med..

[11] Rubinsztein DC, Codogno P, Levine B (2012). Autophagy modulation as a potential therapeutic target for diverse diseases. Nat. Rev. Drug. Discov..

[12] Yun N, Cho HI, Lee SM (2014). Impaired autophagy contributes to hepatocellular damage during ischemia/reperfusion: heme oxygenase-1 as a possible regulator. Free. Radic. Biol. Med..

[13] Ma X (2012). Autophagy is impaired in cardiac ischemia-reperfusion injury. Autophagy.

[14] Amaravadi RK (2007). Autophagy inhibition enhances therapy-induced apoptosis in a Myc-induced model of lymphoma. J. Clin. Invest..

[15] Netea-Maier RT (2016). Modulation of inflammation by autophagy: consequences for human disease. Autophagy.

[16] Cabrera S (2013). ATG4B/autophagin-1 regulates intestinal homeostasis and protects mice from experimental colitis. Autophagy.

[17] Scharl M (2012). Protein tyrosine phosphatase nonreceptor type 2 regulates autophagosome formation in human intestinal cells. Inflamm. Bowel Dis..

[18] Nakahira K (2011). Autophagy proteins regulate innate immune responses by inhibiting the release of mitochondrial DNA mediated by the NALP3 inflammasome. Nat. Immunol..

[19] Yang Z, Klionsky DJ (2010). Mammalian autophagy: core molecular machinery and signaling regulation. Curr. Opin. Cell. Biol..

[20] Travassos LH (2010). Nod1 and Nod2 direct autophagy by recruiting ATG16L1 to the plasma membrane at the site of bacterial entry. Nat. Immunol..

[21] Saitoh T (2008). Loss of the autophagy protein Atg16L1 enhances endotoxin-induced IL-1beta production. Nature.

[22] Nguyen HT (2014). Crohn’s disease-associated adherent invasive Escherichia coli modulate levels of microRNAs in intestinal epithelial cells to reduce autophagy. Gastroenterology.

[23] Ichimura Y (2000). A ubiquitin-like system mediates protein lipidation. Nature.

[24] Li M (2011). Kinetics comparisons of mammalian Atg4 homologues indicate selective preferences toward diverse Atg8 substrates. J. Biol. Chem..

[25] Bartel DP (2009). MicroRNAs: target recognition and regulatory functions. Cell.

[26] Frankel LB (2011). microRNA-101 is a potent inhibitor of autophagy. EMBO J..

[27] Lorenzen JM (2014). MicroRNA-24 antagonism prevents renal ischemia reperfusion injury. J. Am. Soc. Nephrol..

[28] Hinkel R (2013). Inhibition of microRNA-92a protects against ischemia/reperfusion injury in a large-animal model. Circulation.

[29] Korkmaz G (2012). miR-376b controls starvation and mTOR inhibition-related autophagy by targeting ATG4C and BECN1. Autophagy.

[30] Wang JH (2011). Autophagy suppresses age-dependent ischemia and reperfusion injury in livers of mice. Gastroenterology.

[31] Hu J (2016). Targeting TRAF3 signaling protects against hepatic ischemia/reperfusions injury. J. Hepatol..

[32] Feng D (2017). Inhibition of p66Shc-mediated mitochondrial apoptosis via targeting prolyl-isomerase Pin1 attenuates intestinal ischemia/reperfusion injury in rats. Clin. Sci..

[33] Cabrera S (2015). Essential role for the ATG4B protease and autophagy in bleomycin-induced pulmonary fibrosis. Autophagy.

[34] Fougeray S, Pallet N (2015). Mechanisms and biological functions of autophagy in diseased and ageing kidneys. Nat. Rev. Nephrol..

[35] Wang P (2014). Down-regulation of miRNA-30a alleviates cerebral ischemic injury through enhancing beclin 1-mediated autophagy. Neurochem. Res..

[36] Hu R (2014). Complement C5a exacerbates acute lung injury induced through autophagy-mediated alveolar macrophage apoptosis. Cell Death Dis..

[37] Perez-Chanona E, Mühlbauer M, Jobin C (2014). The microbiota protects against ischemia/reperfusion-induced intestinal injury through nucleotide-binding oligomerization domain-containing Protein 2 (NOD2) signaling. Am. J. Pathol..

[38] Zhang X (2014). Cerebral ischemia-reperfusion-induced autophagy protects against neuronal injury by mitochondrial clearance. Autophagy.

[39] Ross SA, Davis CD (2014). The emerging role of microRNAs and nutrition in modulating health and disease. Annu. Rev. Nutr..

[40] Zhu H (2009). Regulation of autophagy by a beclin 1-targeted microRNA, miR-30a, in cancer cells. Autophagy.

[41] Li M (2017). Upregulation of miR-665 promotes apoptosis and colitis in inflammatory bowel disease by repressing the endoplasmic reticulum stress components XBP1 and ORMDL3. Cell Death Dis..

[42] Mariño G, Niso-Santano M, Baehrecke EH, Kroemer G (2014). Self-consumption: the interplay of autophagy and apoptosis. Nat. Rev. Mol. Cell Biol..

[43] Cadwell K (2008). A key role for autophagy and the autophagy gene Atg16l1 in mouse and human intestinal Paneth cells. Nature.

[44] Wittkopf N (2012). Lack of intestinal epithelial atg7 affects paneth cell granule formation but does not compromise immune homeostasis in the gut. Clin. Dev. Immunol..

[45] Mariño G (2010). Autophagy is essential for mouse sense of balance. J. Clin. Invest..

[46] Ma Y, Galluzzi L, Zitvogel L, Kroemer G (2013). Autophagy and cellular immune responses. Immunity.

[47] Zhong Z, Sanchez-Lopez E, Karin M (2016). Autophagy, Inflammation, and immunity: a troika governing cancer and its treatment. Cell.

[48] Cadwell K (2010). Virus-plus-susceptibility gene interaction determines Crohn’s disease gene Atg16L1 phenotypes in intestine. Cell.

[49] Feinberg MW, Moore KJ (2016). MicroRNA regulation of atherosclerosis. Circ. Res..

[50] Putta S (2012). Inhibiting microRNA-192 ameliorates renal fibrosis in diabetic nephropathy. J. Am. Soc. Nephrol..

[51] Hullinger TG (2012). Inhibition of miR-15 protects against cardiac ischemic injury. Circ. Res..

[52] Janssen HL (2013). Treatment of HCV infection by targeting microRNA. N. Engl. J. Med..

[53] Wen S (2017). Necroptosis is a key mediator of enterocytes loss in intestinal ischemia/reperfusion injury. J. Cell. Mol. Med..

[54] Zhang XY (2018). Propofol does not reduce pyroptosis of enterocytes and intestinal epithelial injury after lipopolysaccharide challenge. Dig. Dis. Sci..

[55] Ogasawara M (2017). Suppression of autophagic flux contributes to cardiomyocyte death by activation of necroptotic pathways. J. Mol. Cell. Cardiol..

[56] Pu Q (2017). Atg7 deficiency intensifies inflammasome activation and pyroptosis in pseudomonas sepsis. J. Immunol..

[57] Chiu CJ, McArdle AH, Brown R, Scott HJ, Gurd FN (1970). Intestinal mucosal lesion in low-flow states. I. A morphological, hemodynamic, and metabolic reappraisal. Arch. Surg..

[58] Polishchuk EV (2014). Wilson disease protein ATP7B utilizes lysosomal exocytosis to maintain copper homeostasis. Dev. Cell.

[59] Pei J (2014). Autophagy enhances the replication of classical swine fever virus in vitro. Autophagy.

