# Supplementary material for: Targeting the miR-665-3p-ATG4B-autophagy axis relieves inflammation and apoptosis in intestinal ischemia/reperfusion
Source: Cell Death Dis. 2018 Apr 30;9(5):483. doi: 10.1038/s41419-018-0518-9 (PMC5924757; doi:10.1038/s41419-018-0518-9)
Supplement: Supplementary file 1 — Supplementary Materials and Methods [file 41419_2018_518_MOESM1_ESM.doc]

**Supplementary Data**

**Supplementary Materials and Methods**

**Plasmids**

The Actin-LC3-dNGLUC construct was generated by PCR of hMAP1LC3 (Origene Trueclone AB2841G10) using primers 5’-GACGAATTCATGCCGTCGGAGAAGAC-3’ and 5’-GACGCGGCCGCTTAGGATCCCACTGACAATTTCATCCC-3’ and sub-cloned into the EcoRI and NotI sites of pMOWSdSV. dNGLUC was amplified by PCR and inserted into the BamHI and NotI sites of pMOWSdSV-LC3.

**Luciferase assay**

Cellular ATG4B activity was assayed by measuring the cellular release of an N-terminally deleted form of Gaussia luciferase (dNGLUC), as described by Ketteler, et al^1^. Briefly, Caco-2 cells were seeded in 24-well plates and co-transfected with miR-665-3p and 200 ng of plasmid expressing the appropriate actin-LC3B-dNGLUC (or actin-dNGLUC control) as well as 50 ng of plasmid expressing Firefly luciferase (LUC2, as normalization control). Forty-eight hours later, the medium was collected. The Renilla luciferase and Firefly luciferase assays were carried out according to the manufacturer’s instructions for the dual luciferase reporter assay system and the Firefly luciferase assay system, respectively (Promega, USA).

**Chromatin immunoprecipitation (ChIP) assay**

ChIP assay was performed using the ChIP kit following the manufacturer’s instructions (Abcam, ab500). Briefly, after normoxia or H/R treatment, 2 × 10^6^ Caco-2 cells were fixed with 1% formaldehyde, washed with cold PBS and lysed. Nuclei were digested with micrococcal nuclease to shear crosslinked chromatin, and the lysates were pelleted and precleared. The protein-DNA complexes were incubated with 2 μg rabbit anti-p65 antibody (Cell Signaling Technology, 8242) or a non-specific negative control rabbit IgG overnight and then incubated with protein A/G agarose resin followed by an elution, and cross-links were reversed. After recovery, DNA was subjected to semi-quantitative or quantitative PCR analysis. The specific primers used to amplify the NF-κB-binding region in the promoter of the miR-665-3p gene are indicated in Supplementary Table S4. The inputs were analyzed in parallel using ChIP control primers, which amplify a region of the human β-actin promoter. Fold increases in the binding of p65 to the miR-665-3p gene promoter were quantified by the 2^-ΔΔCT^ method using β-actin DNA as a control.

**Northern blot analysis**

Northern blot was performed as described by Wang, et al^2^. In brief, the samples were run on a 15% polyacrylamide-urea gel, transferred to positively charged nylon membranes (Millipore) followed by crossing-linking through UV irradiation. The membranes were subjected to hybridization with 100 pmol 3’-digoxigenin (DIG)-labeled probe for miR-665-3p overnight at 42℃. The miR-665-3p probe was labeled with DIG using a 3’-End DIG Labeling Kit (Roche). Detection was performed using a DIG luminescent detection kit (MyLab) according to the manufacturer’s instructions. The probe sequence for miR-665-3p was 5’-AGGGGCCUCAGCCUCCUGGU-3’. A DIG-labeled U6 probe was used as an internal control, and its sequence was 5’-TGGAACGCTTCACGAATTTG-3’.

**References**

1. Ketteler, R. & Seed, B. Quantitation of autophagy by luciferase release assay. *Autophagy* **4,** 801-806 (2008).

2. Wang, K. et al. A circular RNA protects the heart from pathological hypertrophy and heart failure by targeting miR-223. *Eur Heart J* **37**, 2602-2611 (2016).
