# Supplementary material for: Targeting the miR-665-3p-ATG4B-autophagy axis relieves inflammation and apoptosis in intestinal ischemia/reperfusion
Source: Cell Death Dis. 2018 Apr 30;9(5):483. doi: 10.1038/s41419-018-0518-9 (PMC5924757; doi:10.1038/s41419-018-0518-9)
Supplement: Supplementary file 2 — Supplementary Figure 1 [file 41419_2018_518_MOESM2_ESM.ppt]

## Slide 1
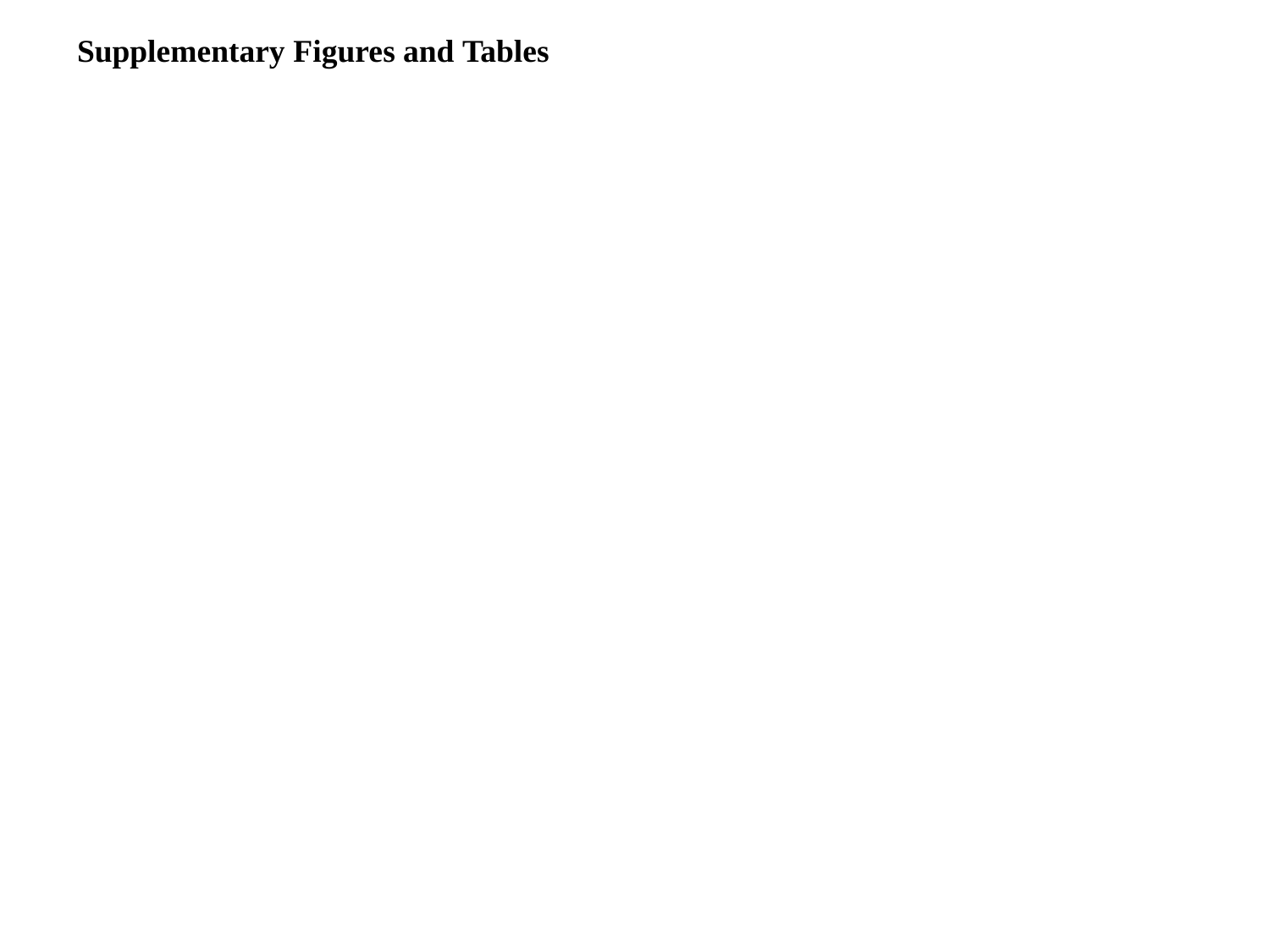

Supplementary Figures and Tables

## Slide 2
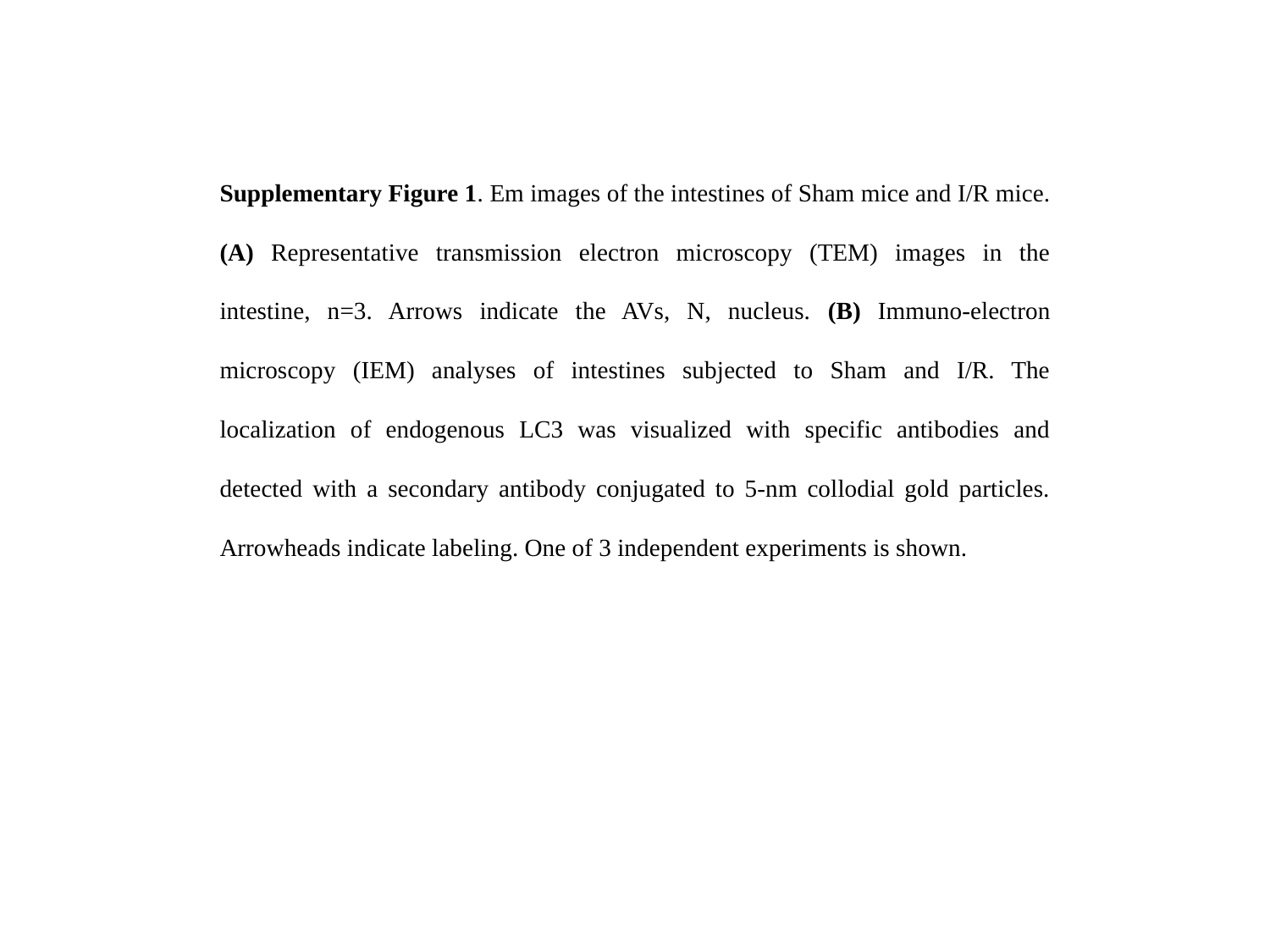

Supplementary Figure 1. Em images of the intestines of Sham mice and I/R mice. (A) Representative transmission electron microscopy (TEM) images in the intestine, n=3. Arrows indicate the AVs, N, nucleus. (B) Immuno-electron microscopy (IEM) analyses of intestines subjected to Sham and I/R. The localization of endogenous LC3 was visualized with specific antibodies and detected with a secondary antibody conjugated to 5-nm collodial gold particles. Arrowheads indicate labeling. One of 3 independent experiments is shown.
