# Supplementary material for: Targeting the miR-665-3p-ATG4B-autophagy axis relieves inflammation and apoptosis in intestinal ischemia/reperfusion
Source: Cell Death Dis. 2018 Apr 30;9(5):483. doi: 10.1038/s41419-018-0518-9 (PMC5924757; doi:10.1038/s41419-018-0518-9)
Supplement: Supplementary file 3 — Supplementary Figure 2-6&Tables [file 41419_2018_518_MOESM3_ESM.doc]

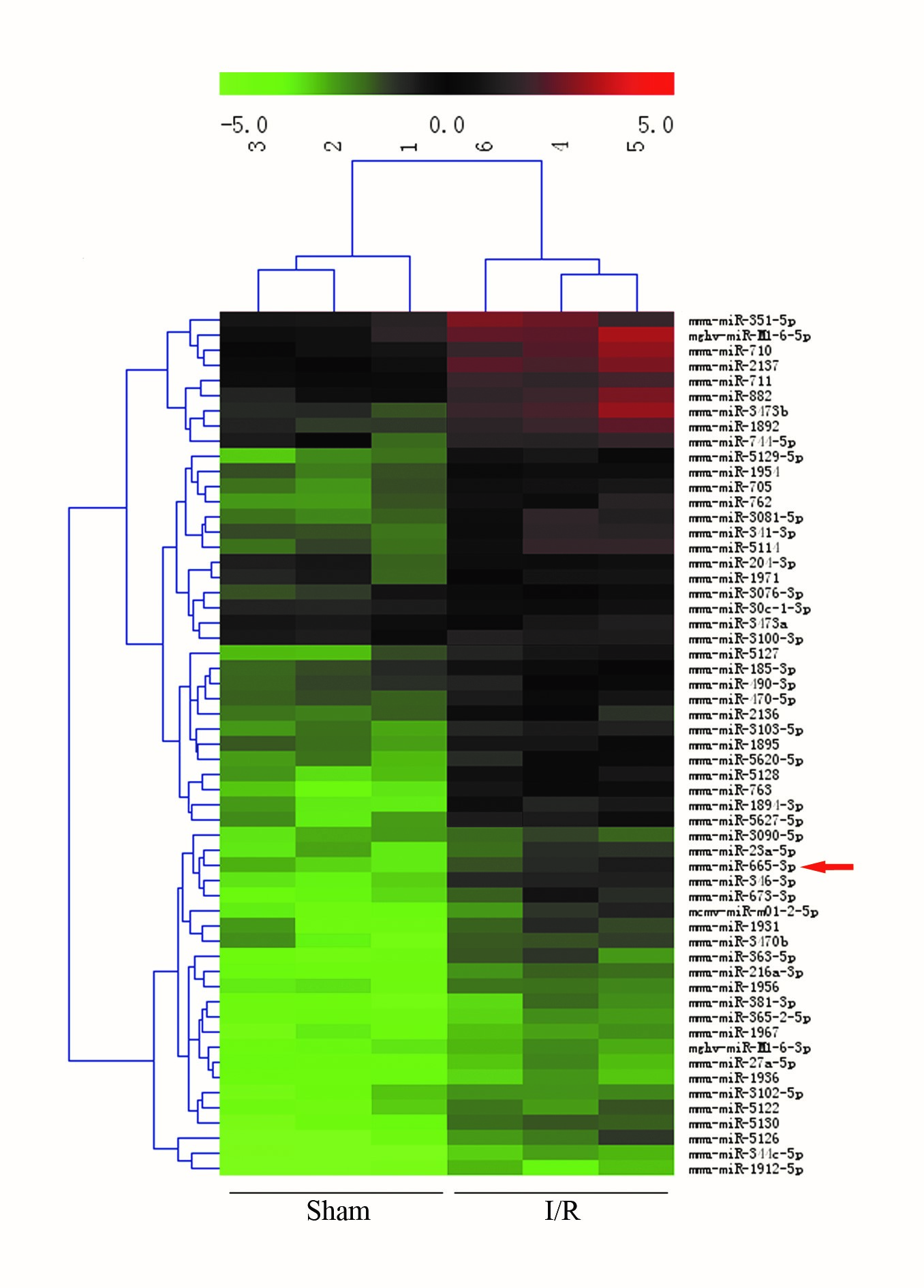


**Supplementary Figure 2**. Differential expression of up-regulated miRNAs in intestinal I/R. Hierarchical clustering analysis of 57 miRNAs that were differentially up-regulated between sham and intestinal I/R samples (greater than 2.0-fold, p < 0.05). The expression levels are represented in shades of red and green, indicating expression above and below the median expression level across all samples (log_2_ scale, from -5.0 to +5.0), respectively.


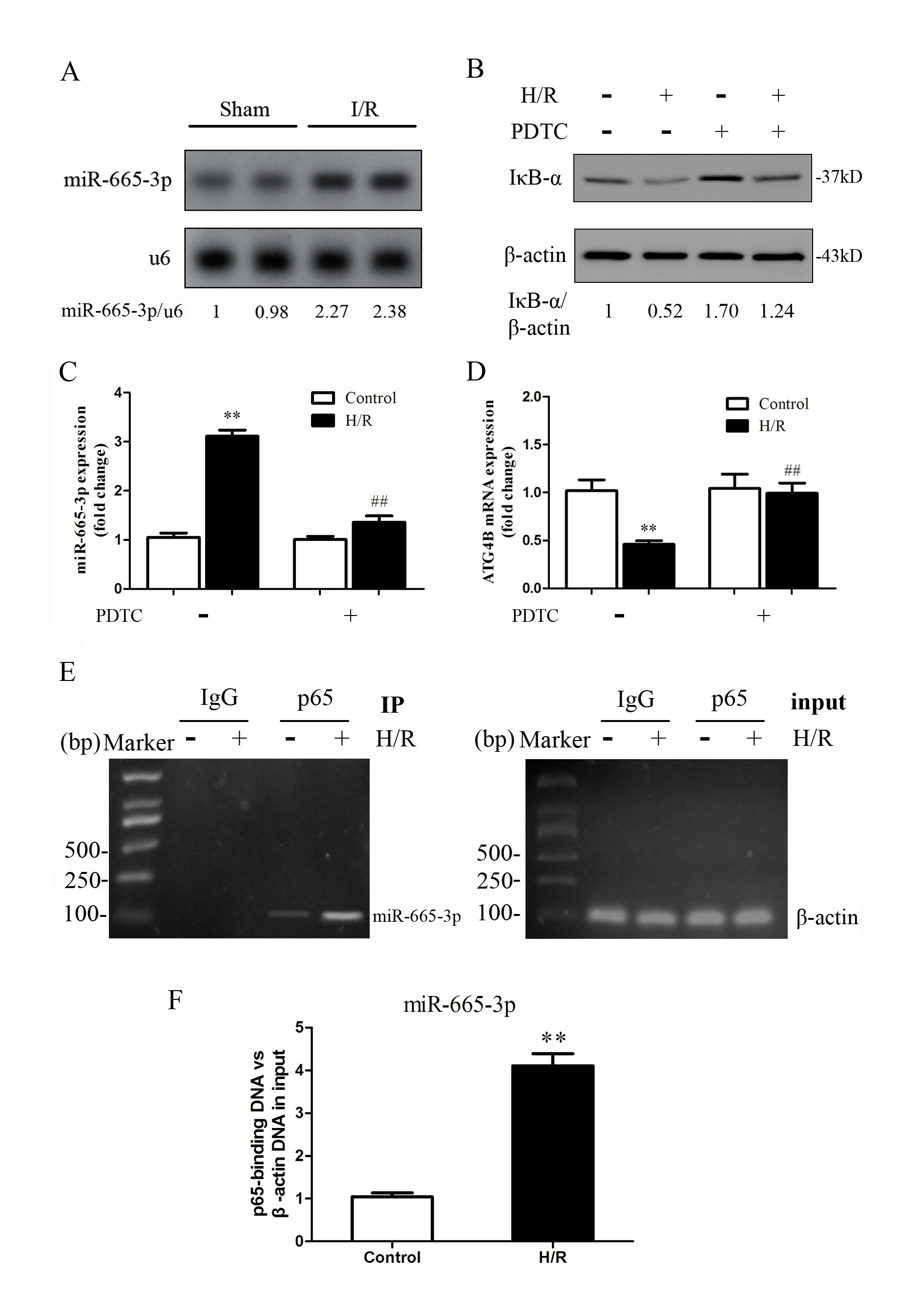


**Supplementary Figure 3**. H/R induces hsa-miR-665-3p upregulation via activating NF-κB pathway. **(A)** Representative northern blots showing miR-665-3p expression in intestine from mice subjected to Sham or I/R conditions. **(B)** Caco-2 cells were treated or not with PDTC (an NF-κB inhibitor) at 100μM for 30 min before H/R. H/R-induced NF-κB activation was assessed by Western blot analysis for IκB-α degradation. **(C-D)** Expressions of miR-665-3p and ATG4B mRNA were quantified by qPCR, n=6. ^**^*P*< 0.01 versus control, ^##^*P*< 0.01 versus H/R. **(E-F)** ChIP analysis for the binding of NF-κB p65 subunit to hsa-miR-665-3p gene promoters. The protein-DNA complexes were immunoprecipitated with anti-p65 or a negative control IgG. **(E)** Representative agarose gels for the p65-binding regions in the hsa-miR-665-3p gene promoters in the IP (immunoprecipitation) and β-actin DNA in the input amplified by semi-quantitative PCR. **(F)** Increase in the binding of p65 to miRNA gene promoters, normalized to β-actin DNA in the input, upon H/R treatment analyzed by qPCR. n=3. ^**^*P*< 0.01 versus control.


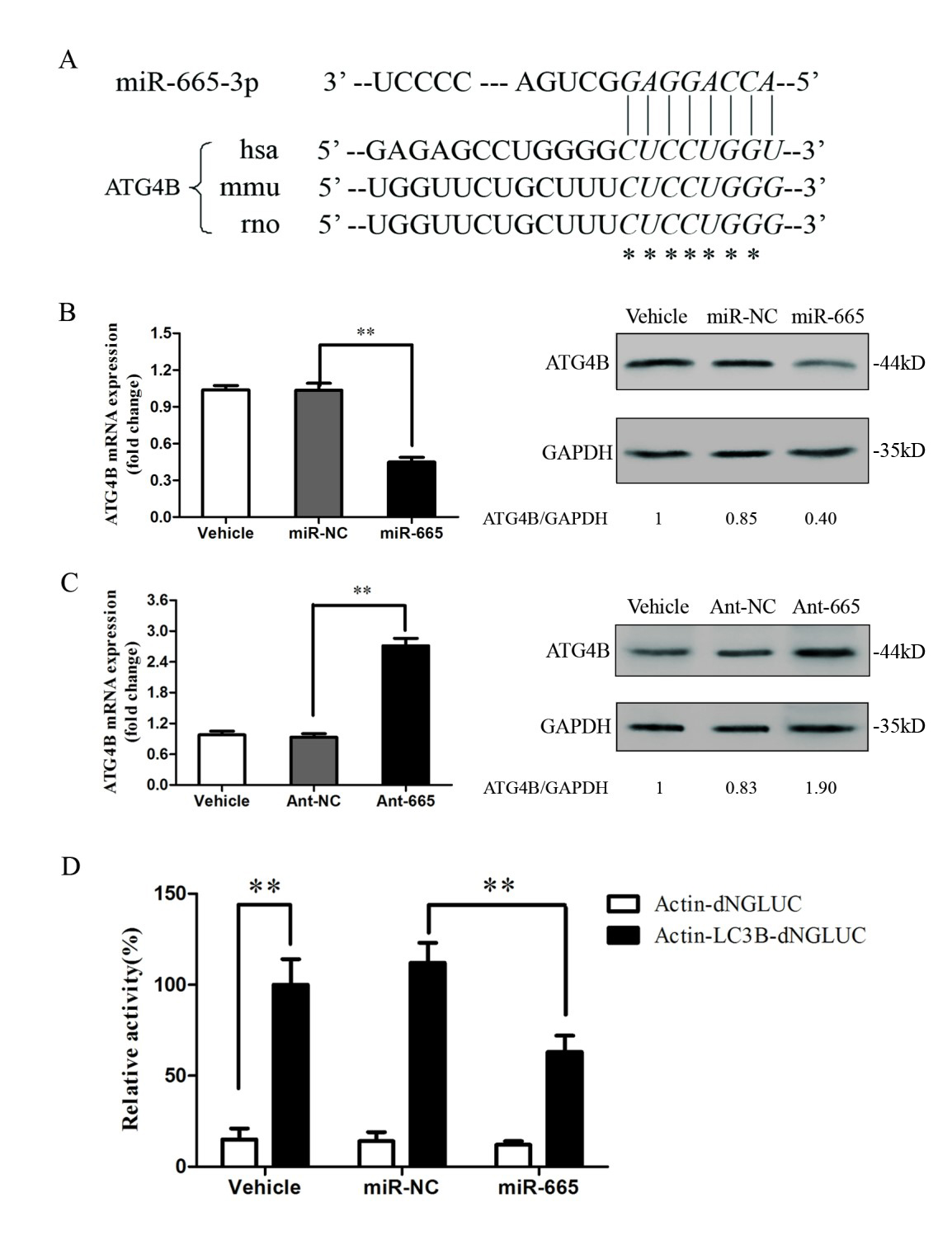


**Supplementary Figure 4**. The effect of rno-miR-665 on ATG4B expression in IEC-6 cells. **(A)** The miR-665 sequence and its predicted binding sites for the human, mouse and rat ATG4B mRNA sequences. **(B-C)** qPCR and western blot of ATG4B in cells treated with agomiR-665-3p and antagomiR-665-3p, n=6, ^**^*P*< 0.01. **(D)** Normalized luciferase activity in culture medium from IEC-6 cells that were co-transfected with plasmids encoding Actin-dNGLUC or Actin-LC3B-dNGLUC together with CMV-Luc2 and miR-NC or miR-665-3p, n=3. ^**^*P*< 0.01.


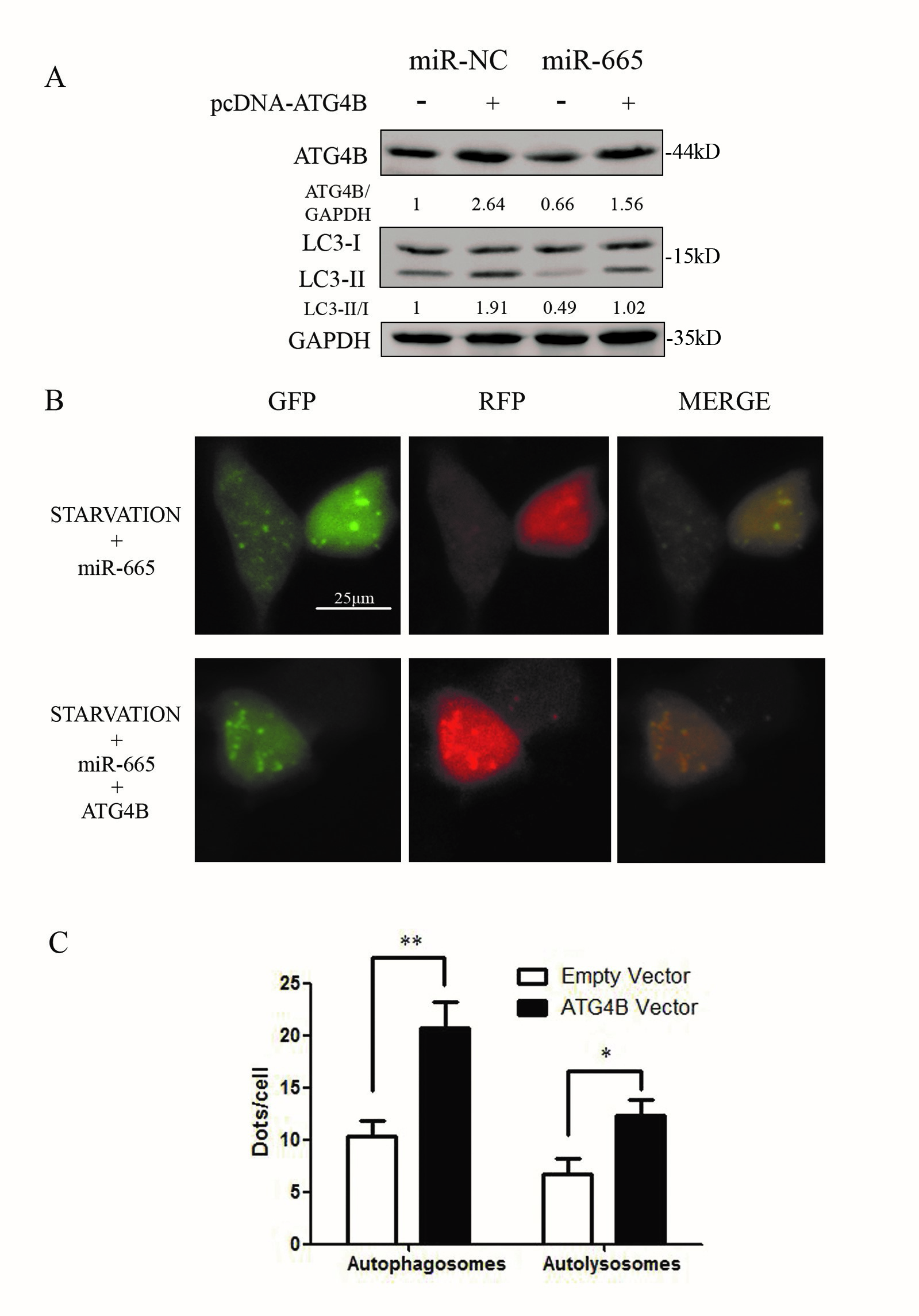


**Supplementary Figure 5.** ATG4B overexpression rescued Caco-2 cells from miR-665-3p-mediated autophagy inhibition. Caco-2 cells were co-transfected with miR-665-3p or miR-NC and an ATG4B expression plasmid. **(A)** Immunoblot analysis of ATG4B and LC3B following the indicated transfections. The ATG4B/GAPDH and LC3B-II/I ratios are shown. **(B)** mRFP-GFP-LC3 dot formation after starvation and transfection with miR-665 and the ATG4B overexpression plasmid. **(C)** Quantitative analysis of autophagosome and autolysosome formation, n=3, ^**^*P*< 0.01 and ^*^*P*< 0.05.


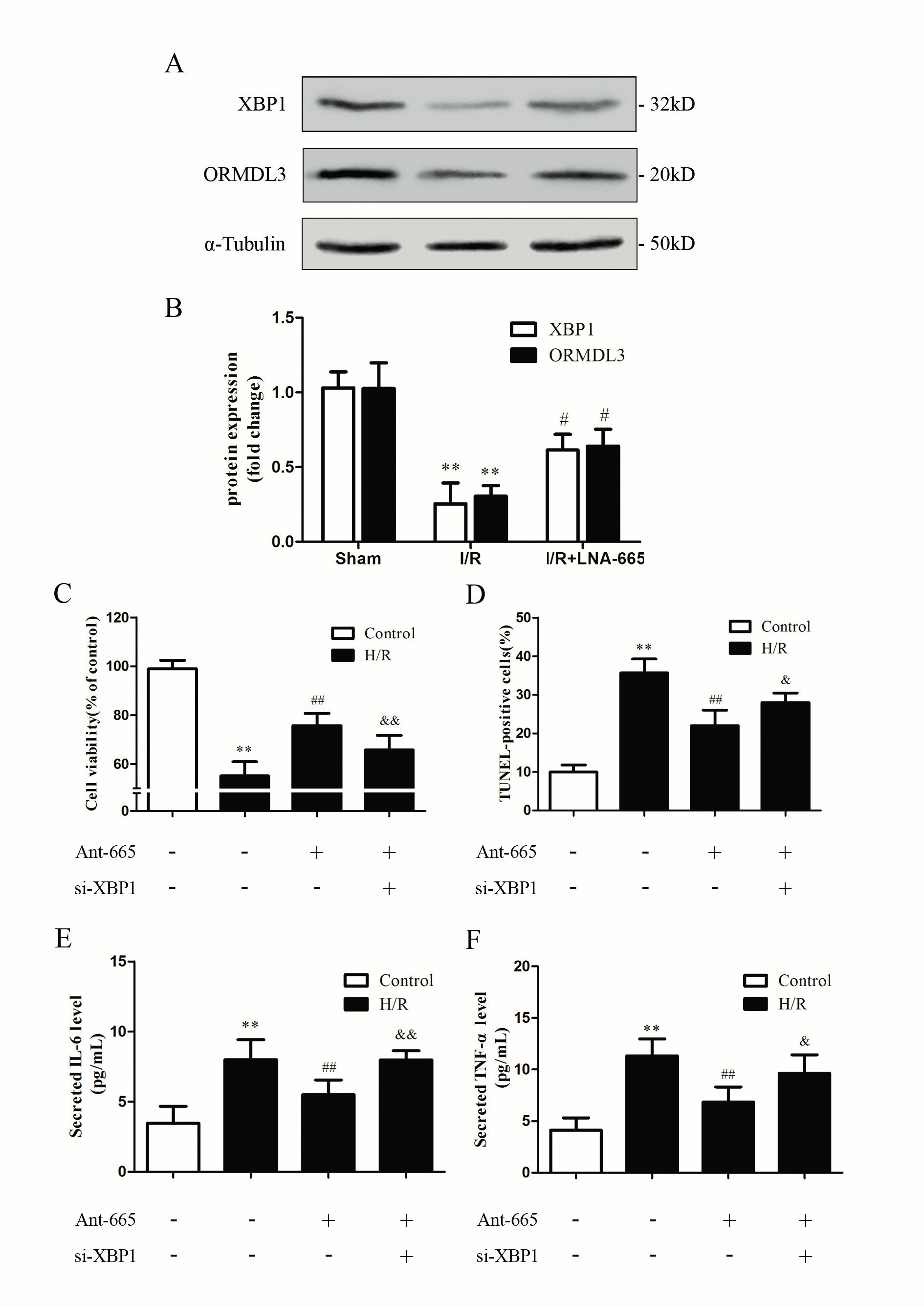


**Supplementary Figure 6.** The expression and function of ER stress-related components (XBP1 and ORMDL3) after miR-665 inhibition in intestinal I/R and cell H/R treatment. Mice were treated with LNA-665 and Caco-2 cells were co-transfected with Ant-NC, Ant-665, and si-XBP1 as indicated. (A-B) Representative immunoblots of XBP1 and ORMDL3 in different groups of intestinal tissues, α-Tubulin served as the loading control. ^**^*P*< 0.01 versus Sham, ^#^*P*< 0.05 versus I/R **(C)** Cell viability was assessed using the CCK-8 assay (n=6). **(D)** Cell apoptosis was evaluated by TUNEL staining assays. **(E-F)** Secreted TNF-α and IL-6 in the cell culture supernatant were quantified by ELISA (n=6). ^**^*P*< 0.01 versus Control, ^##^*P*< 0.01 versus H/R, ^&&^*P*< 0.01 versus H/R+Ant-665.

**Supplementary Table. S1 *In silico* analysis for up-regulated miRNA targeting ATGs during II/R in mouse and human.**

| miRNA | Mouse | | Human | |
| --- | --- | --- | --- | --- |
|  | Target gene | Number of algorithms | Target gene | Number of algorithms |
| miR-763 | ATG7 | 3 | ATG7 | 0 |
| miR-762 | ATG4B | 4 | ATG4B | 1 |
|  | ATG2B | 4 | ATG2B | 0 |
| miR-705 | ATG10 | 5 | ATG10 | 0 |
|  | ATG12 | 5 | ATG12 | 0 |
| miR-882 | ATG2A | 3 | ATG2A | 0 |
|  | ATG2B | 3 | ATG2B | 0 |
| miR-673-3p | ATG4B | 3 | ATG4B | 0 |
| miR-1894-3p | ATG12 | 2 | ATG12 | 0 |
| miR-744-5p | ATG2B | 1 | ATG2B | 2 |
|  | ATG4B | 0 | ATG4B | 4 |
| miR-665-3p | ATG4B | 4 | ATG4B | 4 |
|  | ATG5 | 4 | ATG5 | 0 |
|  | ATG7 | 1 | ATG7 | 1 |
|  | ATG4D | 1 | ATG4D | 4 |
|  | ATG16L1 | 0 | ATG16L1 | 1 |
| miR-381-3p | ATG2B | 4 | ATG2B | 5 |
|  | ATG12 | 1 | ATG12 | 3 |
| miR-185-3p | ATG2A | 4 | ATG2A | 0 |
|  | ATG2B | 4 | ATG2B | 0 |
|  | ATG12 | 3 | ATG12 | 0 |

**Supplementary Table. S2**

**Clinical Characteristics in Patients with Intestinal Infarction**

| **Case** | **Age** | **Sex** | **Diagnosis** | **Gut Ischemia** | **Complication** |
| --- | --- | --- | --- | --- | --- |
| 1 | 56 | Male | acute mesenteric arterial embolism | **4h** | None |
| 2 | 73 | Female | acute mesenteric arterial embolism | **5h** | None |
| 3 | 73 | Male | incarcerated hernia | **4h** | None |
| 4 | 45 | Female | incarcerated hernia | **5h** | None |
| 5 | 67 | Male | strangulated intestinal obstruction | **4h** | None |
| 6 | 66 | Female | strangulated intestinal obstruction | **4h** | None |

**Supplementary Table. S3** Sequences of Agomirs, Antagomirs, LNAs and siRNA

| *Name* | *Sequences (5’-3’)* |
| --- | --- |
| Agomir targeting miR-665-3p | ACCAGGAGGCUGAGGCCCCU  GGGCCUCAGCCUCCUGGUUU |
| Agomir negative control | UUCUCCGAACGUGUCACGUTT  ACGUGACACGUUCGGAGAATT |
| Antagomir targeting miR-665-3p | AGGGGCCUCAGCCUCCUGGU |
| Antagomir negative control | CAGUACUUUUGUGUAGUACAA |
| LNA-665 | GACCTCAGCCTCCTGG |
| LNA-NC | ACGTCTATACGCCCA |
| siRNA targeting ATG4B | GGUGUGGACAGAUGAUCUUTT  AAGAUCAUCUGUCCACACCTT |
| siRNA negative control | UUCUCCGAACGUGUCACGUTT  ACGUGACACGUUCGGAGAATT |
| siRNA targeting XBP1 | CCAGUCAUGUUCUUCAAAU |

**Supplementary Table. S4** Primers for Chromatin immunoprecipitation (ChIP) assay

| Primer | Sequence (5’- 3’) |
| --- | --- |
| miR-665-3p forward | GACAGAAAGAATGAATGGACACA |
| miR-665-3p reverse | GGTGCCCAGAGTGAGATGAA |
